# A SIRT1-centered circuitry regulates breast cancer stemness and metastasis

**DOI:** 10.1038/s41388-018-0370-5

**Published:** 2018-07-23

**Authors:** Lei Shi, Xiaolong Tang, Minxian Qian, Zuojun Liu, Fanbiao Meng, Li Fu, Zimei Wang, Wei-Guo Zhu, Jian-Dong Huang, Zhongjun Zhou, Baohua Liu

**Affiliations:** 1 Guangdong Key Laboratory for Genome Stability and Human Disease Prevention, Shenzhen University Health Science Center, Shenzhen, 518060 China; 2 Medical Research Center (MRC), Shenzhen University Health Science Center, Shenzhen, 518060 China; 3 School of Biomedical Sciences, LKS Faculty of Medicine, the University of Hong Kong, Hong Kong, Hong Kong; 4 Department of Biochemistry & Molecular Biology, Shenzhen University Health Science Center, Shenzhen, 518060 China; 5 Carson International Cancer Center, Shenzhen University Health Science Center, Shenzhen, 518060 China

## Abstract

Cancer stem cell (CSC)-dictated intratumor heterogeneity accounts for the majority of drug-resistance and distant metastases of breast cancers. Here, we identify a SIRT1-PRRX1-KLF4-ALDH1 circuitry, which couples CSCs, chemo-resistance, metastasis and aging. Pro-longevity protein SIRT1 deacetylates and stabilizes the epithelial-to-mesenchymal-transition (EMT) inducer PRRX1, which inhibits the transcription of core stemness factor KLF4. Loss of *SIRT1* destabilizes PRRX1, disinhibits KLF4, and activates the transcription of *ALDH1*, which induces and functionally marks CSCs, resulting in chemo-resistance and metastatic relapse. Clinically, the level of PRRX1 is positively linked to SIRT1, whereas KLF4 is reversely correlated. Importantly, KLF4 inhibitor Kenpaullone sensitizes breast cancer cells and xenograft tumors to Paclitaxel and improves therapeutic effects. Our findings delineate a SIRT1-centered circuitry that regulates CSC origination, and targeting this pathway might be a promising therapeutic strategy.

## Introduction

Tumor is composed of heterogeneous cell populations including those capable of self-renewal and multi-lineage differentiation, termed tumor-initiating cells or cancer stem cells (CSCs) [1]. Accumulating evidence suggests that cancer stemness underlies the majority of drug-/radiation-resistance and recurrent metastases, accounting for more than 90% mortality [2]. CSCs are identified with specific markers, such as CD44 and CD24 (breast CSCs), SSEA-1, ALDH1, and CD133 (lung CSCs) [3–6], the ability to expel DNA dyes, the formation of spheroids, and xenograft tumors [7]. Where and how CSCs are originated has been a focus of cancer research, however, important questions remain [8]. One hypothesis proposes that CSCs are transformed tissue stem cells [9], while others believes that cancer cells acquire stemness via dedifferentiation, sharing the same concept of induced pluripotency [10]. Indeed, key stemness factor OCT3/4, SOX2, KLF4, and NANOG are active in certain tumors [11]. Among the four factors, KLF4 serves in part as an upstream enforcer of feed-forward circuits involving OCT3/4 and SOX2, as well as downstream NANOG. Ectopic expression of KLF4 together with OCT3/4 and SOX2 is sufficient to reprogram somatic cells to pluripotent stem cells (iPSCs). Indeed, KLF4 is activated in diverse human cancers and predictive of mortality [12, 13]; however, the molecular mechanisms by which KLF4 is driven to induce CSCs remain less well documented.

Besides the intratumor genetic and phenotypic heterogeneity, CSCs maintain the plasticity of transition between epithelial and mesenchymal states via a process of epithelial-to-mesenchymal transition (EMT) and the reverse MET during metastasis [14]. Indeed, the restoration of classical EMT transcription factors, such as SNAIL1/2, TWIST1/2, and ZEB1/2, serves as a main mechanism for induced CSCs [15, 16]. However, the relationship between EMT and CSC origination is seemingly controversial. For instance, some studies found EMT rather suppresses tumor-initiating abilities [17], i.e., suppression of PRRX1 promotes MET to enhance stemness and metastatic potential of breast cancers [18]. The mechanisms involved in PRRX1-mediated acquisition of CSC properties remain unknown.

Aging is one major driver of human malignancy [19]. Stem cell number and function drop with aging [20]. However, the molecular mechanisms of such stem cell decline and their relevance to CSC initiation are still puzzled. NAD^+^-dependent deacetylase SIRT1 belongs to the most conserved longevity genes that link metabolism to stress response, endocrine mechanism, aging, and cancer [21]. Here we did two round screens of aging-related genes [22] and identified SIRT1 as a central element regulating age-related CSCs in breast cancer. Depletion of *SIRT1* remarkably increases the number of ALDH1^+^ CSCs, elicits partial MET, and promotes lung metastasis by upregulating KLF4 in human and mouse breast cancer cells. Molecularly, SIRT1 deacetylates and stabilizes PRRX1, an EMT inducer, whose destabilization promotes KLF4 transcription. KLF4 upregulates *ALDH1* transcription and thus induces CSCs. KLF4 inhibitor Kenpaullone overcomes Paclitaxel (PTX) resistance imposed by *SIRT1* deficiency and reduces lung metastasis in mouse models. Our data identify a SIRT1-PRRX1-KLF4-ALDH1 circuitry as a central regulator of CSCs and highlight its therapeutic potential in targeting the progression and metastasis of breast cancer.

## Results

### A SIRT1-centered circuitry underlies age-related CSC induction

**Fig. 1:**
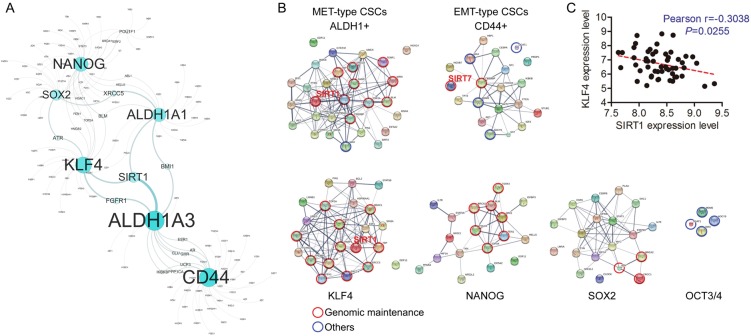
A SIRT1-KLF4-ALDH1A1-ALDH1A3 circuitry dictates age-related breast CSCs. **a** Functionally interacting network modules constructed from genes belonging to Age Human Genes list and stemness-associated genes were analyzed by Prism 5.0 tool (based on Pearson’s correlation coefficient) and visualized in Cytoscape. A graph that nodes have a power law distribution for their number of links, showing correlation between four factors, CSC markers and age-related genes in breast cancer cells (see Table S1). Bigger and darker colored nodes represent proteins with more links. Noted SIRT1 as the node at the tail end of the distribution on the graph. **b** Pathway enrichment analysis of four factors, CSC markers and their associated aging-related proteins by STRING database. Noted a significant enrichment of pathways that safeguard genome integrity, to which KLF4 and ALDH1A1/3 are the most correlated, followed by NANOG, then SOX2, and CD44 and OCT3/4 are the least. CD44 + CSCs are associated with low SIRT7 and share similar set of genes with OCT3/4. **c** Pearson correlation between *SIRT1* and *KLF4* mRNA levels in 52 breast cancer cell lines. *R* is the correlation coefficient

To understand potential links between aging and breast cancer stemness, we employed the GenAge Human Genes list to screen for genes that are correlated with core stemness factor OCT4, SOX2, NANOG, and KLF4 in a cohort of breast cancer cell lines collected from the TCGA database [11, 22, 23]. ALDH1A1/3 and CD44 were included as CSC markers in the analysis. As shown, 75 out of 300 genes are negatively correlated with the core “stemness” program, and 8 of the 75 are reversely correlated with *ALDH1A1/3* or *CD44* (Table S1). Interestingly, the top enrichment list includes *SIRT1*, which is centered in a *KLF4-ALDH1A1-ALDH1A3* triangle of functionally interacting network (Fig. 1a). Further, nine genes in the *OCT4-SOX2-NANOG-KLF4* circuitry and breast cancer CSC markers were analyzed and the correlation was assessed in breast cancer cell lines [11]. A strong reverse correlation between *SIRT1* and *KLF4* was obtained (Fig. 1c and Table S2, *R* *=* −0.304, *P* *=* 0.026, determined by non-parametric Mann–Whitney *t*-test). Of note, a decline of *SIRT7* was associated with high *CD44* (Fig. 1a, b and Table S1, *R* *=* −0.2604, *P* *=* 0.0385). We recently demonstrated that *SIRT7* deficiency activates TGF-β signaling, enhances EMT and promotes lung metastasis [24], and induces mesenchymal-like CSCs marked with CD44^+^CD24^-^ in breast cancers (Figure S1), providing a proof of concept of aging-promoted CSC induction. To confirm the findings, we did pathway enrichment analysis via STRING v10 [25]. The KEGG analysis showed an enrichment of pathways that safeguard genome integrity, wherein KLF4 and ALDH1A1/3 are the most correlated, followed by NANOG, then SOX2, and CD44 and OCT3/4 are the least and even lack of correlation (Fig. 1b and Table S3). Interestingly, EMT-type CSCs (CD44^+^) are associated with low *SIRT7* and share similar set of genes with OCT3/4, suggesting differential roles of Sirtuins on breast cancer stemness: SIRT1 is related to MET-type CSCs (ALDH1^+^), whereas SIRT7 is correlated with EMT-type (CD44^+^). Together, the data points to a SIRT1-KLF4-ALDH1 circuitry, which couples aging and CSCs.

### Genetic ablation of *SIRT1* induces ALDH1^+^ CSCs via upregulating *KLF4*

CSCs are largely responsible for the recurrence and distant metastasis of breast cancer. To investigate the clinical relevance of these CSC-related genes, we analyzed their mRNA levels in cohorts of invasive breast carcinoma (IBC) collected from the Kaplan*–*Meier database [26]. Significantly, two of them, i.e., *SIRT1* and *SIN3A*, are positively correlated with relapse free survival (RFS) and distant metastasis free survival (DMFS) in IBCs (Figure S2A). Further, low *SIRT1* level predicts poor survival, chemotherapy-resistance and metastasis of breast cancer (Figure S2B). We are particularly interested in SIRT1, whose precise function in CSCs remains less well documented.

**Fig. 2:**
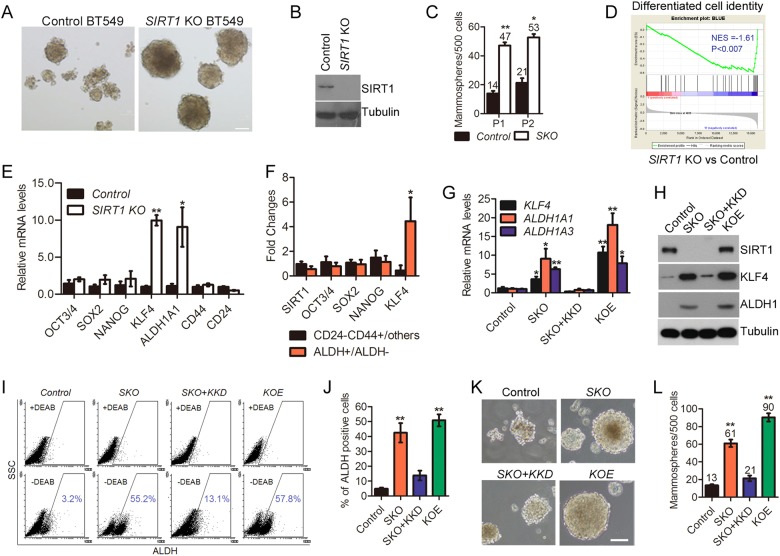
SIRT1 deficiency induces cancer stemness via upregulation of KLF4. **a** Representative images showing mammospheres derived from control and *SIRT1 KO* BT549 cells. Scale bar, 100 µm. **b** Representative Immunoblots showing loss of SIRT1 in *SIRT1 KO* BT549. **c** Quantification of mammosphere-forming efficiency in two passages. Noted that SIRT1 deficiency significantly increased the number of mammospheres. Data represent mean ± SEM from three independent experiments. (*P<0.05, **P<0.01, *t*-test). **d** Gene set enrichment analysis (GSEA) of RNAseq data showing differentiated cell identity signature is reduced in SIRT1-depleted BT549 cells. Normalized enrichment score (NES) and *P* value are shown in the plot. **e** Stemness-related gene expression determined by real-time RT-PCR. The *Y*-axis values are relative fold change for gene transcripts normalized to GAPDH. Data represent mean ± SEM (*n* = 3; *P<0.05, **P<0.01, *t*-test). **f** Levels of *SIRT1* and four stemness core factors in ALDH^+^ Vs ALDH^-^ and CD24^−^CD44^+^ Vs non-CD24^-^CD44^+^ breast cancer cells, assessed by Affymetrix array HU133 Plus 2.0. (*P<0.05, *t*-test). **g**, **h** Stable knockdown of *KLF4* in *SKO* BT549 results in downregulation of ALDH1 on mRNA and protein levels, analyzed by real-time RT-PCR (*n* = 3; * *P*<0.05, ** *P*<0.01, *t*-test) and Western blotting. Reconstituting KLF4 into parental cells elevated ALDH1 expression. **i**, **j** ALDH1 activities of indicated cells were assessed by ALDEFLUOR assay. DEAB was used to establish baseline fluorescence and to define ALDEFLUOR-positive region. Error bars represent SEM (*n* = 3; ** *P*<0.001, *t*-test). (**k**, **l**) Indicated cells were grown in ultra-low attachment surface plates at a density of 500 per well. Assays were conducted after 10 days. **k** Representative images showed typical mammospheres with scale bars (100 µm). **l** Quantification of mammosphere-forming efficiency of parental and mutant cells. The symbol ** indicates *P*<0.01 Vs control groups

To determine its molecular function in CSCs, *SIRT1* was knocked out in triple-negative basal-like breast cancer BT-549 cells via a CRISPR/Cas9 procedure. As predicted, loss of *SIRT1* increased the mammosphere-forming capacity by more than 3 folds (Fig. 2a–c). Similarly, knocking down *Sirt1* in a murine triple-negative basal-like breast cancer cell line 4T1 significantly promoted mammosphere-formation (Figure S3A–C). We next did RNAseq and gene set enrichment analysis (GSEA) in *SIRT1*-deficient and control BT549 cells. As shown, cancer cells with *SIRT1* depletion were positively associated with poor tumor differentiation (NES = −1.61, *P*<0.007) (Fig. 2d). These data implicate that *SIRT1* deficiency promotes CSC-like phenotypes in breast cancer cells.

Having uncovered the role of *SIRT1* deficiency in promoting breast cancer stemness, we then sought to identify its downstream effectors. Consistent with the predicted data (Fig. 1), *SIRT1* KO BT549 cells exhibited increased expression of stemness factor KLF4, coupled with MET-type CSC marker ALDH1A1 (Figure S4A). In contrast, the expression of EMT-type CSC marker CD44 was far from pronounced. Quantitative RT-PCR confirmed that *SIRT1* deficiency increased expression of *KLF4* and *ALDH1A1* in both human BT549 cells and mouse 4T1 cells (Fig. 2e and S3D).

The predicted and experimentally validated co-expression suggest potential molecular crosstalk between KLF4 and ALDH1A1. To test the hypothesis, we first compared the expression of *KLF4, ALDH1A1*, *ALDH1A3*, *CD44*, and *CD24* in human breast cancer samples available in TCGA database [27]. Indeed, the mRNA levels of *KLF4* and *ALDH1A1* are significantly correlated across all samples by Pearson correlation analysis (Figure S4B, *R* *=* 0.3014, *P<*0.0001). The mRNA level of *KLF4* from all 683 samples was also correlated with that of *ALDH1A3* (*R* *=* 0.0915, *P* *=* 0.0168), but not *CD44* or *CD24*. We further analyzed microarray dataset GSE52262 and GSE52327 [4] and found that the mRNA level of *SIRT1* was decreased but that of *KLF4* was elevated in ALDH1^+^ population isolated from human breast cancers (Fig. 2f). In contrast, the negative correlation was not observed in CD44^+^CD24^-^ population. To understand the precise function of KLF4 on ALDH1, we performed online data analysis and found four putative binding sites of KLF4 in the promoter region of *ALDH1A1/3* (Figure S4C). We cloned the promoter regions of *ALDH1A1* and *ALDH1A3* for luciferase assay. As shown, the activity of luciferase driven by both promoters was significantly increased upon HA-KLF4 overexpression (Figure S4D). We further performed quantitative RT-PCR and Western blotting analysis. Knocking down *KLF4* by shRNA in *SIRT1* KO BT549 cells (SKO) downregulated both mRNA and protein levels of *ALDH1*, which were, however, elevated upon ectopic expression of HA-KLF4 (Fig. 2g, h). Consistently, as assessed by ALDEFLUOR assay, the induction of ALDH1^+^ breast CSCs by *SIRT1* deficiency is significantly correlated with *KLF4* level (Fig. 2i, j).

Previous reports identified that ALDH1^+^ population has high proliferative capacity and enhanced mammosphere-forming capacity [28]. Indeed, we found that silencing *KLF4* in SKO cells inhibited both number and size of mammospheres (Fig. 2k, l). On the other hand, KLF4 restoration rescued the proliferative phenotype and mammosphere formation in parental cells (Fig. 2k, l and S4E). Thus, the data support KLF4 as a key CSC-related transcription factor, reversely regulated by SIRT1 in breast tumors.

KLF4 upregulation is likely owing to elevated histone H3 lysine 9 acetylation (H3K9ac) in promoter region caused by SIRT1 removal (Figure S5A). To test the hypothesis, we performed chromatin immunoprecipitation (ChIP) assay in BT549 cells overexpressing SIRT1. However, neither direct binding of SIRT1 or enrichment of H3K9ac was found at the KLF4 promoter region in SKO cells (Figure S5B–C). Therefore, KLF4 is unlikely to be a direct SIRT1 target in our experimental context.

### PRRX1 underlies *SIRT1* deficiency-induced CSCs

**Fig. 3:**
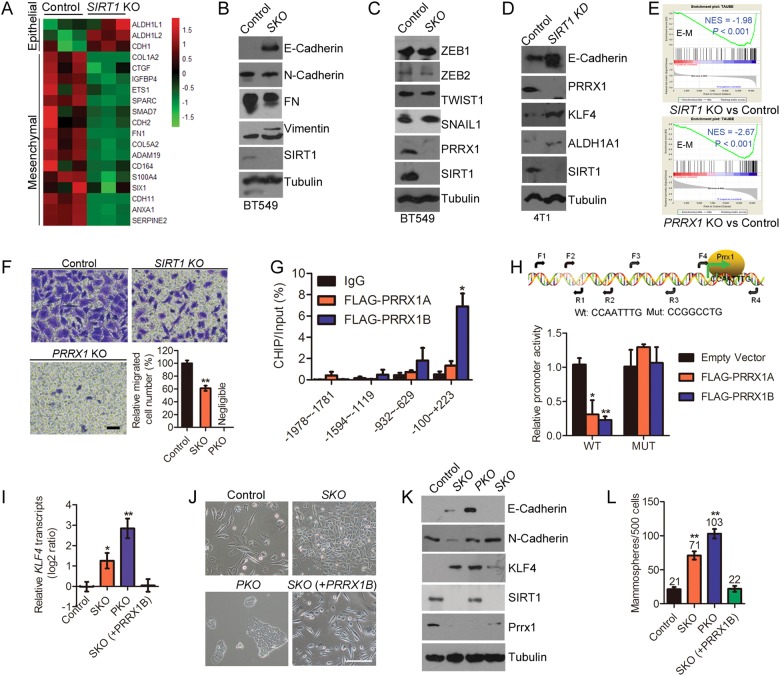
PRRX1 underlies CSCs induced by SIRT1 deficiency. **a** Heat map depicting elevated EMT markers in *SIRT1* KO BT549 compared with control cells. **b**, **c** Immunoblotting of EMT markers (**b**) and inducers (**c**) in Control and SKO BT549. β-Tubulin serves as an internal control. **d** Immunoblotting of E-Cadherin, Klf4, Aldh1a1, Prrx1 and Sirt1 in Control and *Sirt1* KD 4T1 cells. **e** GSEA showing reduced EMT signatures in *SIRT1*-depleted and *PRRX1*-depleted BT549 cells. Normalized enrichment score (NES) and *P* value are shown in the plot. **f** Representative photos of cell invasion by Boyden chamber method analysis. Scale bar, 50 µm. Right lower panel, quantification of the percentage of invaded cells. Data represent mean ± SEM (*n* = 4; ** *P*<0.01, *t*-test). **g** CHIP-qPCR assay using anti FLAG antibody or control IgG in HEK293 cells transfected with FLAG-tagged PRRX1A or PRRX1B plasmid, showing the strong DNA binding of PRRX1B on the *KLF4* promoter region (*n* = 3; * *P*<0.05, *t*-test). **h** Luciferase assays with HEK293T cells co-transfected with empty vector, PRRX1A or PRRX1B constructs together with indicated *KLF4* promoter reporter together with control vectors (*n* = 3; * *P*<0.05, *t*-test). **i** Relative *KLF4* mRNA transcripts in wild-type BT549 cells and indicated mutants detected by quantitative RT-PCR. Data represent mean ± SEM (*n* = 3; * *P*<0.05, ** *P*<0.01, *t*-test). **j** Representative morphologic photos of indicated cells. Scale bar, 100 µm. **k** Western blotting analysis of E-Cadherin, N-Cadherin, KLF4, PRRX1, and SIRT1 in indicated cells. Re-introduction of PRRX1 in SKO cells partially restored EMT and reduced KLF4 level. **l** Mammosphere formation under suspension culture conditions was elevated in indicated cell lines. Data are represented as mean ± SEM (*n* = 3; ** *P*<0.01, *t*-test)

In initial stage of somatic reprogramming, KLF4 activates MET. Moreover, silencing *SIRT1* induces epithelial-like ALDH1^+^ CSCs in BT549 breast cancer cells, which are basal-like and originally enriched with mesenchymal traits. Therefore, we reasoned that the EMT-MET program might be involved in *SIRT1* deficiency-induced CSCs. To test the hypothesis, we compared the RNAseq data (GSE112365) of control and SKO cells, and found a significant downregulation of EMT program in SKO cells (Fig. 3a). Further, a partial MET was evidenced by the restoration of membranous E-Cadherin and the reduction of Fibronectin in *SIRT1* KO BT549 cells (Fig. 3b). EMT-MET program is regulated by a group of transcription factor ZEB1/2, TWIST1, SNAIL1, SLUG, and PRRX1. However, quantitative RT-PCR analysis in BT549 and 4T1 cells revealed that silencing *SIRT1* by either CRISPR/Cas9 or shRNA barely altered the mRNA levels of these EMT transcription factors (Figure S6 and S3E). Instead, the protein level of PRRX1 was found decreased in SKO cells (Fig. 3c). Knocking down *SIRT1* downregulated the level of PRRX1 but upregulated that of E-Cadherin, KLF4 and ALDH1A1 in mouse 4T1 cells (Fig. 3d).

We then asked whether PRRX1 regulates CSCs via the SIRT1-KLF4-ALDH1 axis. To this end, TRANSFAC and JASPAR databases were employed to search for potential transcriptional regulatory elements in *KLF4* promoter. Interestingly, a PRRX1-binding motif was identified (Figure S7A), suggesting that the decline of PRRX1 most likely underlines the MET transition and CSC phenotypes induced by *SIRT1* deficiency. We thus generated *PRRX1* KO (PKO) BT549 cells via CRISPR/Cas9 system and examined the mesenchymal and epithelial traits. Similar to SKO, depletion of *PRRX1* induced an epithelial shift, abrogated mesenchymal traits and inhibited cell invasion in PKO cells (Fig. 3e, f).

PRRX1 has two isoforms, PRRX1A and PRRX1B, which share identical N-terminus (a.a. 1–199) but different C-terminus (Figure S7B). We found that *PRRX1B* was much more highly expressed than *PRRX1A* in BT549 cells (Figure S7C). Further determined by ChIP-qPCR analysis, FLAG-PRRX1B rather than FLAG-PRRX1A was enriched at a −100 to +223 region of *KLF4* promoter, suggesting potential binding motifs (Fig. 3g). Next, the ~1.3 kb *KLF4* promoter region containing the predicted PRRX1-binding core sequence TCGGCCAATTT was cloned and subjected for luciferase reporter assay. As shown, the activity of *KLF4* promoter-driven luciferase was significantly reduced upon FLAG-PRRX1 transfection (Fig. 3h). In addition, a 4-bp mutation (AATT to GGCC) in the seed sequence restored PRRX1-suppressed luciferase activity. These data suggest that PRRX1 binds to the TCGGCCAATTT motif locating in the downstream of *KLF4* transcription start site, and block its transcription.

To determine whether PRRX1 regulates *KLF4* transcriptipn in vivo, we re-introduced the abundant isoform PRRX1B into SKO cells and compared *KLF4* mRNA transcripts. A quantitative assessment revealed that loss of *SIRT1* or *PRRX1* markedly increased the mRNA level of *KLF4*, and re-introduction of PRRX1B completely restored *KLF4* transcripts to levels close to those in parental cells (Fig. 3i). Next, we examined the correlation of SIRT1, KLF4, and PRRX1 in EMT transition and sphere forming abilities. Depletion of *SIRT1* or *PRRX1* induced distinct morphological change from spindle-like to cobblestone-like appearance, which was completely reversed by ectopic PRRX1B (Fig. 3j). Both SKO and PKO cells showed mixed epithelial/mesenchymal traits, i.e., a partial MET. Restoration of PRRX1 in SKO cells reversed MET, i.e., upregulation of N-Cadherin but loss of E-Cadherin, accompanied with a reduction of KLF4 (Fig. 3k). Compared with control BT549 cells, we observed 3.4-fold increase and 4.9-fold increase in the number of mammospheres in *SIRT1*-depleted and *PRRX1*-depleted cells, respectively (Fig. 3l). Notably, the mammosphere-forming capability of SKO cells with ectopic *PRRX1B* was comparable to that of parental controls, likely due to direct suppression of *KLF4* transcription. Together, the data implicate that PRRX1 underlies *SIRT1* deficiency-induced CSCs.

### SIRT1 deacetylates PRRX1

**Fig. 4:**
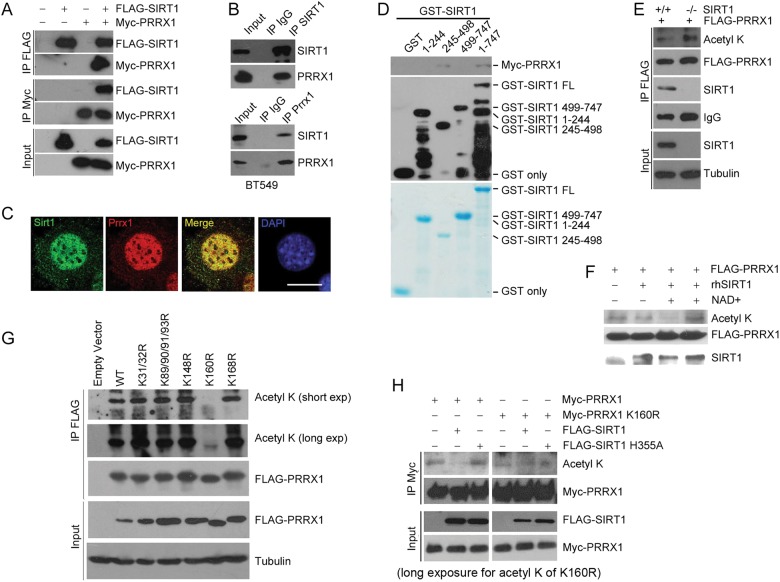
SIRT1 interacts with and deacetylates PRRX1 at K160. **a** HEK293T cells were transfected FLAG-SIRT1 and/or Myc-PRRX1 plasmids. Whole-cell lysates were subjected to IP with FLAG antibody (top) or Myc antibody (bottom) followed by immunoblotting with anti-FLAG and anti-Myc antibodies. **b** Endogenous interaction of SIRT1 and PRRX1 was detected in BT549 cells by IP using SIRT1 antibody (top) or PRRX1 antibody (bottom) followed by immunoblotting with anti-SIRT1 and anti-PRRX1 antibodies. **c** Immunofluorescence staining showing co-localization of SIRT1 and PRRX1 proteins in the nucleus of mouse fibroblasts. Scale bar, 20 µm. **d** GST pull-down assay showing the interaction between GST-SIRT1 fragments and Myc/His-PRRX1. **e** Sirt1-/- MEFs were transfected with the indicated constructs, and FLAG-PRRX1 acetylation was determined by Western blotting analysis. **f** FLAG-PRRX1 acetylation level in the presence or absence of rhSIRT1, NAD+ and Sirtuin specific inhibitor NAM were measured by in vitro acetylation assay and detected by Western blotting. **g** Lysine (**k**) acetylation of various Myc-PRRX1 mutants. Noted that K160R almost completely abolished the acetylation of Myc-PRRX1. **h** Immuno-purified wild-type or catalytic-dead HY mutant FLAG-SIRT1 was used to perform in vitro deacetylation assay. Acetylation of lysine residues was detected with anti-pan-Ac-lysine antibody. Noted that K160R almost completely abolished the acetylation of Myc-PRRX1

SIRT1 is a NAD^+^-dependent deacetylase regulating various proteins. We hypothesized that SIRT1 might deacetylate PRRX1. As determined by co-immunoprecipitation (Co-IP), the binding between SIRT1 and PRRX1 was consistently observed (Fig. 4a, b). The interaction was confirmed by fluorescence microscopy displaying co-localization of SIRT1 and PRRX1 in MEFs (Fig. 4c), and co-localized GFP-SIRT1 and DsRed-PRRX1 in HEK293 cells (Figure S8). To determine specific domains that mediate such interaction, GST-tagged full-length SIRT1 (GST-SIRT1^FL^), N-terminal SIRT1 (GST-SIRT1 ^1–244^), enzymatic domain of SIRT1 (GST-SIRT1^245–498^), and C-terminal SIRT1 (GST-SIRT1^499–747^) were expressed in bacteria, purified and incubated with Myc-PRRX1. Myc-PRRX1 was pull down by GST-SIRT1^FL^ and GST-SIRT1^245–498^ but not GST-SIRT1^1–244^, GST-SIRT1^499–747^ or GST (Fig. 4d), supporting a direct interaction between PRRX1 and SIRT1.

We next examined whether SIRT1 deacetylates PRRX1. As shown, the acetylation level of PRRX1 was inhibited by a pan-sirtuin inhibitor nicotinamide (NAM) and a SIRT1-specific inhibitor EX527, but not class I/II histone deacetylase (HDAC) inhibitor TSA (Figure S9A). To test whether SIRT1 deacetylates PRRX1, FLAG-PRRX1 constructs were transfected into *Sirt1*^*+/+*^ and *Sirt1*^*-/-*^ MEFs, followed by immunoprecipitation using antibody against FLAG to determine acetylation level of FLAG-PRRX1. As expected, *SIRT1* depletion led to a significant increase in acetylated FLAG-PRRX1 (Fig. 4e). Cells treated with specific siRNA against *SIRT1* showed a significant increase in the acetylation level of FLAG-PRRX1 compared with scramble (Figure S9B). Direct deacetylation of PRXX1 by SIRT1 was evaluated using an in vitro deacetylation assay, wherein purified protein FLAG-PRRX1 was incubated with recombinant human SIRT1 (rhSIRT1) and its acetylation level was determined. As shown, SIRT1 deacetylated PRRX1 in an NAD^+^-dependent manner, and that was abolished by NAM, a pan-Sirtuin inhibitor (Fig. 4f). To determine the lysine residues that are deacetylated by SIRT1, we performed site-directed mutagenesis, mutating lysine (K) to arginine (R) to mimic nonacetylated form, i.e., K31/32R, K89/90/91/93R, K148R, K160R, and K166R. As shown, the acetylation of FLAG-PRRX1 was completely abolished in K160R mutant, while others were barely affected (Fig. 4g). Overexpression of SIRT1 decreased the acetylation level of Myc-PRRX1, but enzyme-dead mutant H355A loosed such ability (Fig. 4h). In addition, neither WT nor H355 A SIRT1affected the acetylation level of K160R mutant. These results suggest that K160 is a deacetylation site directly regulated by SIRT1.

### SIRT1-mediated deacetylation prevents proteasomal degradation of PRRX1

**Fig. 5:**
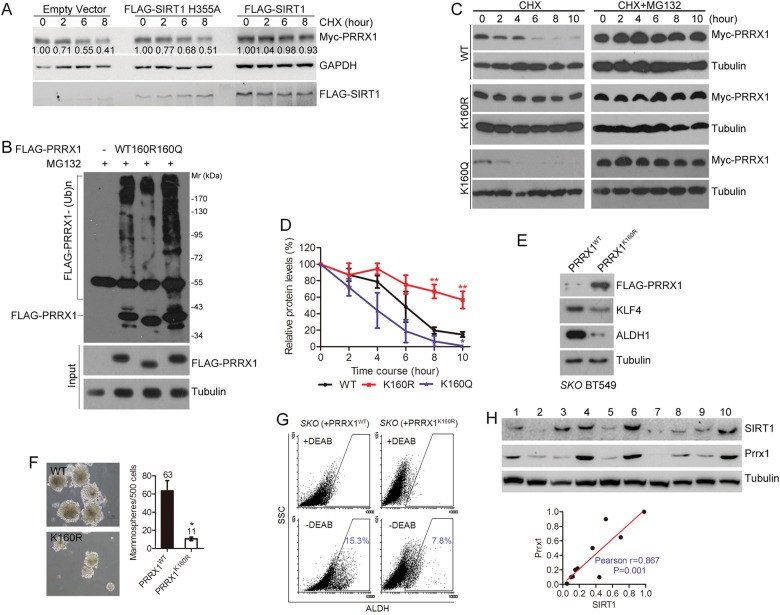
SIRT1 stabilizes PRRX1 by inhibiting ubiquitination. **a** Protein levels of Myc-PRRX1 in HEK293T cells expressing empty vector, FLAG-SIRT1 or FLAG-SIRT1 H355A in the presence of CHX. Relative intensity was quantified by ImageJ^®^. **b** Hypoacetylation-mimicking K160R inhibits while the hyperacetylation-mimicking K160Q promotes ubiquitination. **c** Protein levels of Myc-PRRX1, Myc-PRRX1^K160R^ and Myc-PRRX1^K160Q^ in the presence (right) or absence of MG132 (left). (**d**) Quantification of protein levels in the presence of CHX. Data are presented as mean ± SEM (*n* = 4; * *P*<0.05, K160Q Vs WT; ** *P*<0.01, K160R Vs WT). Noted that the hypoacetylation-mimicking K160R inhibited PRRX1 degradation. In contrast, the hyperacetylation-mimicking K160Q accelerated the degradation. **e** Western blotting analysis of KLF4 and ALDH1 levels in SKO BT549 cells with ectopic FLAG-PRRX1 WT or K160R mutant. **f** Mammosphere formation in SKO BT549 harboring ectopic FLAG-PRRX1^WT^ or FLAG-PRRX1^K160R^. Left, representative photos are shown. Scale bar, 100 µm. (*n* = 3; *P<0.05, *t*-test). Right panel, quantification of mammospheres. Data are presented as mean ± SEM (*n* = 3; * *P*<0.05, *t*-test). **g** ALDEFLUOR assay in SKO BT549 cells with ectopic FLAG-PRRX1 WT or K160R mutant in the presence or absence of ALDH inhibitor DEAB. **h** Upper panels, Western blotting analysis of Sirt1 and Prrx1 protein levels in MMTV-PyMT tumors. Lower panels, a positive correlation between Sirt1 and Prrx1 was found

Given only protein level was affected, it is most likely that *SIRT1* deficiency-mediated PRRX1 decline is attributable to accelerated protein degradation. Indeed, the treatment with proteasome inhibitor MG132 reversed the degradation of endogenous PRRX1 in the presence of protein synthesis inhibitor cycloheximide (CHX) (Figure S9C), suggesting that SIRT1 regulates PRRX1 protein stability via ubiquitination-proteasome system. To test it, FLAG-PRRX1-expressing HEK293 cells were treated with EX527, NAM, or TSA, and the protein levels were examined. In the presence of CHX, both EX-527 and NAM destabilize FLAG-PRRX1, whereas TSA merely affected it (Figure S9D). To determine whether SIRT1 activity is essential for PRRX1 stability, we co-transfected wild-type and enzyme-dead H355A SIRT1 with Myc-PRRX1 construct to HEK293 cells, treated the cells with CHX, and then monitored the decline of Myc-PRRX1 by Western blotting. As shown, the degradation rate of Myc-PRRX1 was reduced in the presence of SIRT1 instead of H355A (Fig. 5a), suggesting that SIRT1-mediated deacetylation prevents the proteasome degradation of PRRX1.

To address how SIRT1 increases PRRX1 protein stability, we examined ubiquitination of WT, K160R and K160Q PRRX1. SIRT1 might deacetylate PRRX1 at K160 to inhibit preferential attachment for ubiquitin. As shown, poly-ubiquitination of PRRX1 K160R was largely impaired, whereas that of K160Q was enhanced (Fig. 5b). Consistently, K160R inhibited but K160Q accelerated the protein degradation of Myc-PRRX1. The degradation of Myc-PRRX1 was rescued by MG132, indicating that it is, at least partially, subjected to proteasomal degradation (Fig. 5c). The half-life of Myc-PRRX1 WT, K160R, and K160Q are around 5.4 ± 0.3, 14.7 ± 3.0 and 4.9 ± 0.3 h, respectively (Fig. 5d). Together, the data implicate that SIRT1 deacetylates PPRX1 to inhibit its polyubiquitination and proteasomal degradation.

Given that PRRX1 deficiency promotes MET and CSCs in breast cancer [18], we reasoned that re-introduction of PRRX1 would reverse MET and CSC-like phenotypes in *SIRT1*-depleted cells. To test it, WT and K160R PRRX1 were reconstituted in SKO cells. As shown, reconstitution of K160R suppresses KLF4 and ALDH1 expression (Fig. 5e), and abolished the mammosphere formation (Fig. 5f), accompanied with deceased ALDH activity (Fig. 5g). To evaluate the in vivo relevance, we explored a MMTV-PyMT transgenic model of breast cancer and isolated primary mammary tumors for Western blotting analysis (Fig. 5h). Quantitative data confirmed a strong positive correlation between endogenous SIRT1 and PRRX1 levels in breast tumors (*R* = 0.867, *P* = 0.001). Collectively, our data support the notion that PRRX1 instability, at least partially, underlies *SIRT1* deficiency-induced epithelial plasticity and CSC properties of breast cancers.

### Suppressing KLF4 sensitizes breast cancer cells to chemotherapy

**Fig. 6:**
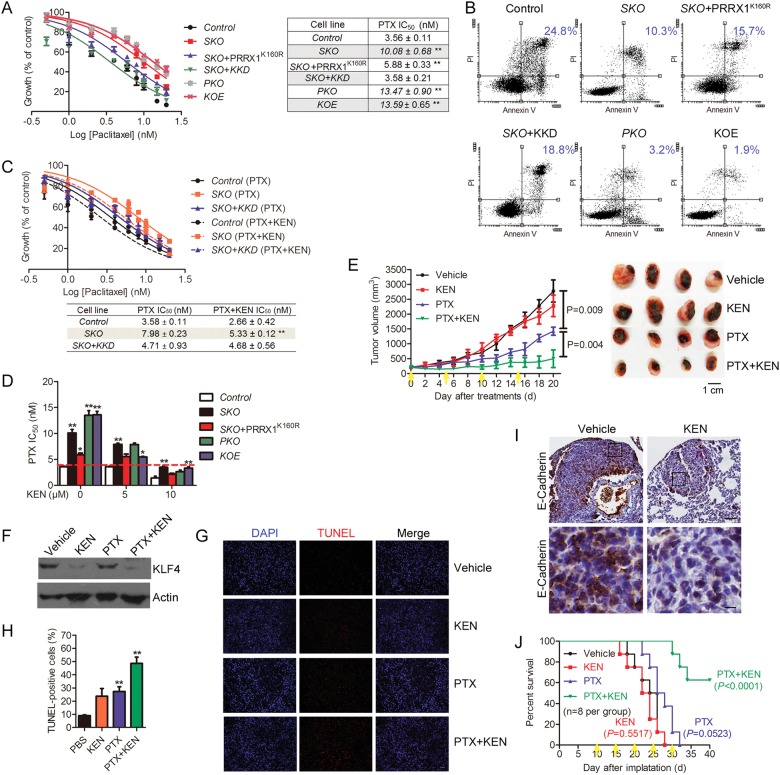
Suppressing KLF4 attenuates *SIRT1* depletion-induced chemoresistance. **a** Dose response curves and IC_50_ of unmodified and indicated mutant cells treated with PTX. (*n* = 3; ** *P*<0.01, *t*-test). **b** Apoptotic analysis assessed by Annexin-V/PI staining in cells after 24-h treatment with 10 nM PTX. **c** Log-dose response analysis of Control, SKO and SKO + KKD BT549 cells with PTX or PTX plus with 5 µg KEN. (*n* = 3; ** *P*<0.01, *t*-test). **d** IC50 of the indicated cells treated with PTX in the presence or absence of KEN. (*n* = 3; * *P*<0.05, ** *P*<0.01, *t*-test). **e** 4T1 cells (10^5^ cells per mouse) were subcutaneously injected into nude mice. Ten days after injection, mice were injected intratumorally with vehicle (0.2% Tween 80/PBS), PTX (25 mg kg^−1^ body weight) and PTX plus 200 µg KEN per mouse every five days. Left, tumor volumes were calculated and plotted (mean ± SEM, *n* = 4). Right, representative tumors collected from the different treatment groups of mice. **f** Detection of KLF4 in vehicle, PTX alone and PTX + KEN treated tumors by Western blotting analysis. **g** Cell death was examined by TUNEL labeling primary tumors from vehicle, KEN alone, PTX alone and PTX + KEN treated mice. Scale bars, 100 μm. **h** Percentages of TUNEL positive cells in indicated treatments. Data are presented as mean ± SEM (*n* = 3,** *P*<0.01, *t*-test). **i** Representative micrographs illustrating E-Cadherin expression in pulmonary metastases from mice treated with KEN or vehicle alone. Scale bar: upper panel, 100 µm and lower panel, 10 μm. **j** Mice were treated vehicle, PTX (25 mg kg^−1^ body weight) and PTX plus 200 µg KEN per mouse (*n* = 8 per group) on day 10, 15, 20, and 25. Cumulative survival of mice with indicated treatments (Kaplan–Meier, with log-rand test)

CSCs accounts for the majority of drug-resistance of breast cancer; the SIRT1-PRRX1-KLF4 core circuitry might regulate such chemo-resistance. We evaluated the impact of *SIRT1* deficiency or *KLF4* overexpression on apoptosis or cell survival in response to Paclitaxel (PTX), which belongs to the most frequently applied cytotoxics. However, almost half of subjects develop resistance after first treatment [29]. Here, log-dose curves and IC50 values of control and various mutant cells were determined by MTT assay. Depletion of *SIRT1*, *PRRX1* or re-introduction of KLF4 in BT549 cells rendered the cells more resistant to apoptosis; the PTX resistance caused by *SIRT1* deficiency was reversed by ectopic expression of PRRX1 K160R or silencing *KLF4* (KKD) (Fig. 6a). This is consistent with the apoptotic data as adjudged by Annexin V/PI staining (Fig. 6b).

We reasoned that combination of chemotherapy and KLF4 inhibitors like Kenpaullone (KEN) might have a synergetic effect on killing breast cancer cells. In particular, log-dose response analysis showed a KLF4-dependent cytotoxicity of KEN in BT549 cells (Fig. 6c). Adding non-cytotoxic concentration of KEN significantly sensitized breast cancer cells to PTX with minimal IC50 (Fig. 6d). Given the excellent tolerability of KEN, we assessed the effect of combined treatment with PTX and KEN on the highly aggressive and multidrug-resistant murine 4T1 cells. As shown, PTX alone decreased the tumor size, while PTX plus KEN almost abrogated the tumor formation (Fig. 6e). Consistent with in vitro cultured BT549 cells, co-treatment with PTX and KEN not only downregulated KLF4 level but also induced cell death (1.8-fold increase of TUNEL-positive cells relative to PTX only group) in 4T1 xenografts (Fig. 6f–h). Histological examination revealed that lung metastatic lesions derived from KEN-treated mice was smaller than those received vehicle (Fig. 6i), with strong membranous staining of E-cadherin. Further, while PTX alone had a modest effect on the survival of 4T1 and SKO BT549-bearing mice, PTX plus KEN administration resulted in significantly improved survival (Fig. 6j and S10A). Mice treated with vehicle or PTX alone often died from lung metastases (Fig. 6i and S10B-C). Together, pharmacological intervention of KLF4 elicits strong therapeutic effect on chemo-resistance induced by *SIRT1* or *PRRX1* deficiency.

### The SIRT1-PRRX1-KLF4 core circuitry controls breast cancer metastasis

**Fig. 7:**
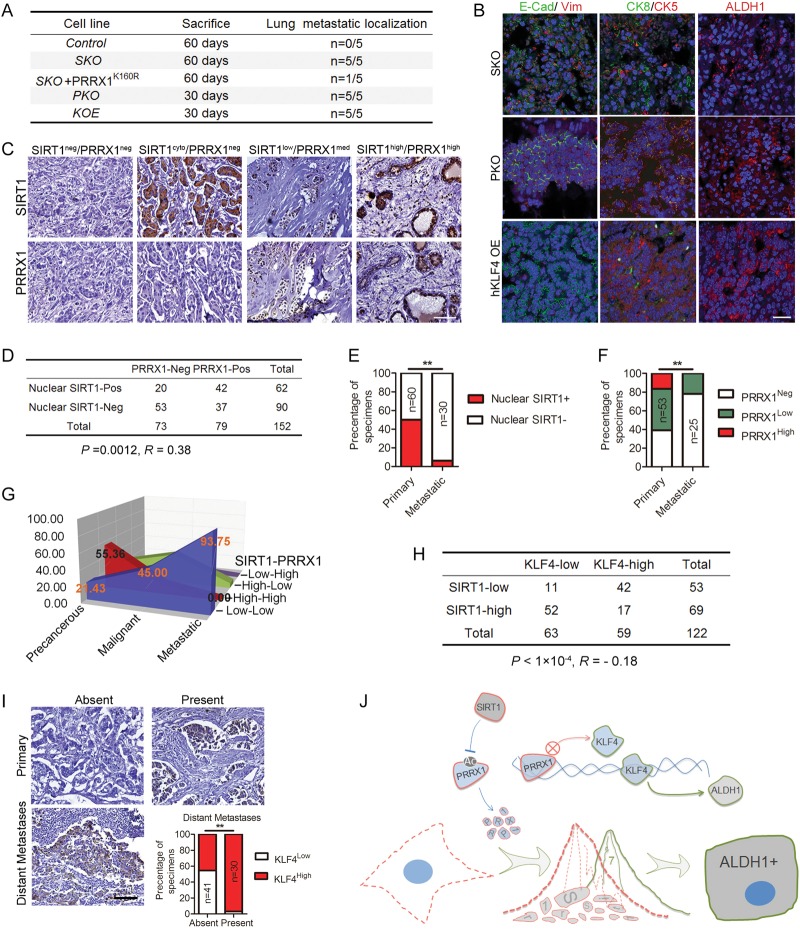
The SIRT1-PRRX1-KLF4 axis regulates breast cancer metastasis. **a** Silencing *PRRX1* or forced expression of *KLF4* increases metastatic capacity of non-tumorigenic basal-like BT549 cells. **b** Immunofluorescence staining of E-Cadherin, CK5, CK8, and ALDH1 in lung metastases. Scale bar, 50 µm. **c** Immunohistochemistry analysis of SIRT1 and PRRX1 in consecutive sections of a human breast cancer array. Of note, PRRX1 was predominantly localized in the nucleus (right and middle right), and SIRT1 was found in both nucleus (right and middle right) and cytoplasm (middle left). Scale bar, 100 µm. **d** Correlation between SIRT1 and PRRX1 protein levels in human breast tumors. Statistical significance was determined by a χ2 test. *R* is the correlation coefficient. **e** Percent specimens that are SIRT1-negative-in-nucleus according to clinical parameter of malignant tissues and lymph node metastases (** *P*<0.001, χ2 test). **f** Percent specimens with high expression (++/+++; red columns) and low expression (+, green columns) and absence (−, white columns) of PRRX1 according to clinical parameter of malignant tissues and lymph node metastases (** *P*<0.001, χ2 test). **g** Percent SIRT1-PRRX1 axis levels in indicated tissues. Of note, almost 94% lymph node metastases were stained with low-low SIRT1-PRRX1 (*P*<0.001). **h** Correlation between SIRT1 and KLF4 levels in human breast tumors. Statistical significance was determined by a χ2 test. *R* is the correlation coefficient. **i** Immunohistochemistry analysis of KLF4 in malignant tissues with or without distant metastases and lymph node metastases. Scale bar, 100 µm. Lower panel, percent of specimens with high expression (++/+++; red columns) and low expression (−/+, white columns) of KLF4 according to the distant metastases (** *P*<0.001, χ2 test). **j** A working model for SIRT1-PRRX1-KLF4-ALDH1 circuitry. SIRT1 deacetylates and thus stabilizes PRRX1; PRRX1 inhibits the transcription of KLF4; KLF4 activates transcription of ALDH1, which induces and marks CSCs

Spatiotemporal heterogeneity caused by EMT-MET transition and CSCs also underlies cancer metastasis [30]. The partial MET and cancer stemness induced by *SIRT1* deficiency, and the correlation between SIRT1 and DMFS prompted us to investigate the function of SIRT1-PRRX1-KLF4 axis in breast cancer metastasis. To this end, 5 × 10^6^ parental and various mutant BT549 cells were injected intravenously into nude mice. As shown, the lungs of all five mice were colonized by SKO cells within 60 days, and PKO and KLF4-overexpressing BT549 cells formed micro-metastases within 30 days (Fig. 7a). Significantly, overexpression of PRRX1 K160R suppressed lung metastasis in SKO cells: only 1 out of 5 mice formed metastatic lesions. Almost no metastatic lesions were detected in all mice injected with unmodified BT549 cells, supporting an essential role of the SIRT1-PRRX1-KLF4 axis in regulating breast cancer metastasis. Further, Immunofluorescence staining demonstrated that these metastases were positive for E-Cadherin and ALDH1 (Fig. 7b), highlighting the significance of MET-type CSCs in breast cancer metastasis. Interestingly, while some tumor cells in the metastatic lesions of SKO cells still retained keratin 5 (CK5), an undifferentiated marker of basal lineage, some cells in these lesions activated expression of keratin 8 (CK8), a marker of luminal cells. These results suggest that loss of *SIRT1* induces dedifferentiation and increases cellular heterogeneity. In the metastatic lesions of PKO or KOE cells, keratins 5 and 8 were co-expressed (Fig. 7b), indicating that these cells are immediate progenitors.

To further investigate the clinical relevance of the SIRT1-PRRX1-KLF4 axis, human breast cancer arrays were subjected for immunohistochemical microscopy analysis. The overall clinicopathologic parameters were summarized in Figure S11A-C. While SIRT1 was found in both nucleus and cytoplasm, PRRX1 was predominantly nuclear (Fig. 7c). A significant positive correlation (*R* *=* 0.38, *P* = 0.0012) between nuclear SIRT1 and PRRX1 was observed in breast carcinomas (Fig. 7d). Considering the nuclear proportion of SIRT1 might be more relevant in regulating PRRX1, we evaluated the correlation between nuclear SIRT1 and breast malignancy. In this case, 50% (60 of 120) malignant tissues and 94% (30 of 32) metastases were nuclear SIRT1 negative/low (Fig. 7e), suggesting a reverse correlation between nuclear SIRT1 and breast cancer progression. Likewise, 40% (27 of 68) invasive ductal carcinoma, 68% (15 of 22) invasive lobular carcinoma and more than 94% (30 of 32) metastases were completely negative for PRRX1 (Fig. 7f), whereas 90% (27 of 30) ductal/lobular carcinoma in situ and fibroadenoma were positive. Importantly, the nuclear proportion of SIRT1 and PRRX1 are significantly correlated with each other, i.e., more than 90% metastases were nuclear SIRT1-PRRX1 double negative/low (Fig. 7g, *P*<0.001), supporting a SIRT1-PRRX1 axis in regulating breast cancer metastasis.

We next did immunohistochemical analysis of KLF4 on 122 subjects of breast cancer to validate the association between KLF4 and SIRT1 and to elucidate the clinical relevance of KLF4 overexpression in patients with distal metastases. As shown, a significant reverse correlation (*R* *=* −0.18, *P*<1 × 10^−4^) between KLF4 and SIRT1 was observed in these specimens, wherein 79% (42 of 53) of the tumors with low SIRT1 exhibited high KLF4 expression, and 75% (52 of 69) of the tumors with high SIRT1 exhibited low KLF4 (Fig. 7h). In breast cancer patients, decrease of SIRT1 was associated with reduced PRRX1 but increased KLF4 (Figure S11D), and the latter was positively correlated with distal metastases (*P*<0.001, χ2 test) (Fig. 7i).

Collectively, the data suggest that SIRT1 blocks the transition between epithelial and mesenchymal state of breast cancer cells. It elicits such function via deacetylating and stabilizing the EMT inducer PRRX1, which inhibits the transcription of core stemness factor KLF4. Loss of *SIRT1* destabilizes PRRX1 and leads to upregulation of KLF4, accompanied with partial MET. KLF4 subsequently promotes transcription of *ALDH1*, which induces and marks CSCs, and clinically leads to chemo-resistance and metastatic relapse (Fig. 7j).

## Discussion

Independent studies found that the reprogramming factor OCT3/4, SOX2, NANOG, and KLF4 induce CSCs in human mammary epithelial cells and promote carcinogenesis in vivo [31, 32]. Among these factors, KLF4 functions to enhance OCT3/4 and SOX2. Here, we performed a bioinformatics screening in a list of aging-related genes and identified a SIRT1-KLF4-ALDH1 circuitry driving CSC origination. This process is mediated by an EMT inducer PRRX1 and accompanied with partial MET. Indeed, the sequential activation of EMT-MET enhances efficiency of somatic reprogramming [33, 34], and is also critical for tumor initiation, progression, and metastasis [14]. In breast cancer, EMT-type CSCs with high CD44 but low CD24 in the invasive front and MET-type CSCs expressing high level of ALDH1 in the tumor interior co-exist [4]. Dynamic epithelial and mesenchymal transition might dictate different pluripotent stages of CSCs, which are coordinated by a series of EMT inducers. Several types of carcinoma cells have been found to gain tumor-initiating capability, usually depicted as the defining trait of CSCs, after induction of EMT [16, 35]. Yet other studies report the presence of proposed MET-type CSCs via downregulating PRRX1, rather than carcinoma cells that undergo an EMT [14, 18]. The roles and underlying mechanism for PRRX1 in cancer pathogenesis and the origination of MET-type CSCs remain unknown.

In this study, we revealed a novel SIRT1-PRRX1-KLF4 circuitry, whereby SIRT1 deacetylates and thus prevents the proteasomal degradation of PRRX1.We demonstrate that PRRX1 exerts its stemness-promoting effect on breast cancer cells by orchestrating the crosstalk between epithelial and proliferative states via transcriptional regulation of *KLF4*. Loss of PRRX1 disinhibits *KLF4* transcription, which activates functional breast CSC marker ALDH1A1 and ALDH1A3. *SIRT1* depletion enhances epithelial plasticity in basal-like breast cancer cells, induces highly proliferative MET-type ALDH1^+^ CSCs, confers cellular resistance to chemotherapy and promotes distant metastases.

Increasing proofs support a partial MET or hybrid EMT/MET in cancer metastasis [36]. Here, an upregulation of epithelial E-Cadherin but reduced mesenchymal Fibronectin were shown, while other mesenchymal traits like N-Cadherin and Vimentin remained unchanged. Only epithelial like ALDH1-positive cells were present in lung metastases, suggesting the contributing role of epithelial plasticity in metastatic localization in the context of *SIRT1* or *PRRX1* deficiency or KLF4 overexpression. This is consistent with a recent report revealing the coexistence of epithelial and mesenchymal traits in primary breast tumors, but only epithelial cells metastasize to murine lung [37].

The role of SIRT1 in breast malignancy and metastasis has been controversial [38]. SIRT1 was found elevated in leukemia, skin cancer, prostate cancer, and colon cancer [39–42], but decreased in colorectal adenocarcinoma [43]. In contrast, most studies in transgenic models support a tumor-suppressing role of SIRT1 as no increased tumor formation has been documented so far [44, 45]. In this study, if nuclear and cytoplasmic proportions are considered, 52% cases of malignant or metastatic breast cancers were SIRT1 negative or low. This is indeed consistent with the public data that 20–50% cases were SIRT1 negative/low [46–49]. As a transcription factor, PRRX1 is predominantly localized in the nucleus. Given that PRRX1 directly interacts with SIRT1, the nuclear proportion of SIRT1 seems more relevant in the SIRT1-PRRX1 axis. Remarkably, nuclear staining of the SIRT1-PRRX1 axis were absent/low in more than 45% cases of IBC and 90% lymph node metastases, thus providing potential diagnostic and therapeutic targets for human breast malignancies. In line with our data, a predominant cytoplasmic localization of SIRT1 has been reported in a group of cancer cells, owing to aberrant PI3K/IGF-1R signaling [50]. It would be worthwhile to investigate whether PI3K/IGF-1R signaling is affected in human breast cancers in future study, especially those showing increased cytoplasmic but reduced nuclear level of SIRT1. Nonetheless, our findings emphasize the significance of protein subcellular distribution in cancer biology, diagnosis and targeted therapy, and explain the seeming discordance between SIRT1 and human breast cancer in the literature. Further, as it works as key regulator in the SIRT1-PRRX1-KLF4 axis, PRRX1 likely plays a more dominant role in breast cancers. Indeed, we found that PRRX1 declines along tumor progression and is stained negative in 78% metastases, making it alone a potential diagnostic biomarker for breast cancer. Collectively, we propose that depleting *SIRT1* accelerates the degradation of PRRX1 and disinhibits *KLF4* transcription, leading to a partial MET, ALDH1-positive CSCs, and distant metastases. Reduced nuclear level of SIRT1-PRRX1 axis is positively correlated with lung metastasis of breast cancer. The findings not only shed light on the understanding of roles for SIRT1 in regulating breast malignancy, but also indicate a unique mechanism of CSC origination, malignant transformation, and metastasis.

## Materials and Methods

### Cell lines, constructs and oligos

Human BT-549 breast cancer cell line was obtained from American Type Culture Collection (ATCC), and is free of mycoplasma contamination. HEK293T cells and mouse embryonic fibroblasts (MEFs) were maintained in Gibco^®^ DMEM (Life Technologies) supplemented with 10% FBS (Biosera America), 1% non-essential amino acid (Life Technologies) and 0.1 mM 2-mercaptoethanol (Sigma-Aldrich). BT549 cells were cultured in Gibco^®^ RPMI 1640 (Life Technologies) supplemented with 10% FBS. For mammosphere formation, single BT-549 cells were plated in MammoCult^®^ Medium (Stem Cell Technologies), at a density of 2 × 10^3^ cells/cm^2^. After a 10-day culture, mammospheres larger than 150 μm in diameter were photographed and counted. Cells were treated with MG132 (Sigma-Aldrich), Cycloheximide (CHX) (Sigma-Aldrich), Trichostatin A (TSA) (Sigma-Aldrich), Nicotinamide (NAM) (Sigma-Aldrich), Ex-527 (Sigma-Aldrich), Paclitaxel (PTX) (Sigma-Aldrich), or Kenpaullone (KEN) (Sigma-Aldrich) for the indicated time at various concentration.

SIRT1 (h) and Sirt1 (m) siRNA oligos were purchased from Life Technologies. EGFP-SIRT1 construct was a gift from Dr Qiwei Zhai (Shanghai Institutes for Biological Sciences, China). FLAG-SIRT1 construct was from Dr. Zhenkun Lou (Mayo Clinic College of Medicine, USA). pcDNA3.1-HA-KLF4 full length construct was purchased from Addgene (Plasmid #34593). Full-length PRRX1A and PRRX1B were amplified from human cDNA using PrimeSTAR^®^ GXL DNA Polymerase (Takara) and cloned into pDsRed1-Express-C1 and pET28a (+) (Clontech^®^). Full-length PRRX1 ORF with 3 × FLAGs was cloned into pcDNA3.1 (+)/Myc-his (Life Technologies) through NheI and HindIII cutting sites. The K31/32 R, K89/90/91/93 R, K148R, K160R, K160Q, and K166R mutants were introduced into full-length PRRX1B by PCR-driven overlap extension using the two PCR primer pairs shown in Table S4. ~1.2 kb KLF4 promoter region (WT and mutant) were amplified from BT549 genomic DNA using PrimeSTAR^®^ GXL DNA Polymerase (Takara) and cloned into pDsRed1-Express-C1 and pET28a (+) (Clontech^®^). F Digestion enzymes were from New England Biolabs.

Normal transfection was performed with FuGENE^®^ 6 Transfection Reagent (Promega) and Opti-MEM^®^ medium (Life Technologies) according to the manufacturer’s instructions. siRNA transfection was performed with Lipofectamine^®^ RNAiMAX Transfection Reagent (Life Technologies). Drug-resistant colonies were picked and expanded to establish stable cell lines.

### Animals

Six-week-old female athymic *nu/nu* mice were purchased from Vital River Company. Animals were randomly assigned to the treatment group by simple randomization, and investigator was single blinded during group allocation. Mice were housed and handled in accordance with protocols approved by the Committee on the Use of Live Animals in Teaching and Research of Shenzhen University.

### CRISPR/Cas9-mediated genome editing

The CRISPR/Cas9-mediated gene mutagenesis was conducted as described [51]. Briefly, pX459 vector (Addgene#48139) was digested with BbsI and ligated with annealed oligonucleotides. BT-549 cells were transfected with pX459-gSIRT1 or pX459-gPRRX1 using Lipofetamine^®^ 3000 Transfection Reagent according to manufacturer’s introductions. After transfection, cells were selected with 1 μg/ml puromycin (Invitrogen). The targeted mutation(s) were confirmed by DNA sequencing (BGI) and western blot analysis.

### Immunofluorescence staining

After sample preparation by fixation, permeabilization, and blocking, the slides were incubated with primary antibody diluted in 1% BSA/PBST at 4 °C overnight. Following primary antibody incubation, the slides were then washed twice with PBST and incubated with conjugated secondary antibodies (Life Technologies) for 1 h at room temperature. Following secondary antibody incubation, the slides were washed three times with PBST and cover-slipped with SlowFade^®^ Gold antifade reagent with DAPI (Life Technologies). Immunostained samples were imaged using Zeiss LSM710 confocal microscopes and analyzed with the Zen software. Photos were processed with Photoshop CS^®^.

### Cell viability and apoptosis assays

Cell viability assays were performed using MTT dye (3-(4, 5-Dimethylthiazol-2-yl)-2, 5-diphenyltetrazolium bromide) method. Relative cell numbers were determined by staining with MTT dye at indicated time point, dissolving the stained cell with SDS-Dimethylformamide solution, and measuring with a spectrometer (OD590). Apoptosis was measured by flow cytometry with the FITC Annexin-V Apoptois Detection Kit (BioVision) according to the manufacturer’s instruction. Annexin V^+^PI^–^ and Annexin V^+^PI^+^ cells were considered early and late apoptotic cells, respectively.

### ALDH FACS

ALDEFLUOR Kit (Stem Cell Technologies) was used for the identification of ALDH high and low expression cells according to manufacturer’s protocol.

### Protein extraction, Western blotting, and Co-immunoprecipitation

Lysates from cells in culture were prepared by suspending in suspension buffer (0.1 M NaCl, 10 mM Tris-HCl [pH 7.5], 1 mM EDTA [pH 8.0], 1 mM DTT and protease inhibitors) and a same volume of Laemmli buffer (0.1 M Tris-HCl [pH 7.0], 4% SDS, 20% glycerol, 1 mM DTT and protease inhibitors), and boiled for 10 min. Western blotting was performed according to standard procedures and quantified by Image J^®^. For statistical analysis, at least three independent immunoblots were analyzed. For co-immunoprecipitation, cells were lysed into cold RIPA 250 buffer (50 mM Tris-HCl [pH 7.4], 250 mM NaCl, 0.5% NonidetP-40, 1 mM EDTA, 1 mM DTT, 10 mM NAM and protease inhibitor cocktail). 2 μg primary antibodies or appropriate control IgGs (Santa Cruz) were added to the lysates and incubated for 2 h on a rocking platform at 4^o^C, following incubation with Protein G Agarose beads (Life Technologies) for overnight. The beads were then washed twice with lysis buffer and boiled in SDS sample buffer. Subsequently, the protein suspension was collected and detected by western blotting.

### Glutathione S-Transferase (GST) pull-down assay

GST or GST fusion proteins was expressed in Rosetta (DE3) induced by 0.4 mM isopropyl-β-D-thio-galactoside. The pellet of bacteria was resuspended in TEN buffer (20 mM Tris-HCl [pH 7.4], 0.1 mM EDTA, and 100 mM NaCl) supplemented with 1 mM DTT and protease inhibitor cocktail and sonicated. The resulting soluble fractions were incubated with Glutathione Sepharose^®^ 4 fast flow (GE Healthcare) and then washed twice. Purified Myc/His-Prrx1 protein was expressed in bacteria, purified and incubated with GST or GST fusion proteins. After incubation at 4℃ for 4 h with rotation, the beads were washed four times with TENT buffer (0.5% Nonidet P-40, 20 mM Tris-HCl [pH 7.4], 0.1 mM EDTA, and 300 mM NaCl) and analyzed by western blotting with anti-Myc or anti-GST antibody.

### RNA isolation, reverse transcription, and quantitative PCR analysis

Total RNA was extracted using TRIZOL^®^ reagent (Life Technologies). Complementary DNA was synthesized with 2 μg of total RNA by using SuperScript^®^ III First-Strand Synthesis System (Life Technologies). Quantitative PCR was performed with SYBR^®^ Ex Taq Premixes from Takara on an ABI StepOne plus system (Applied BioSystems). Gene specific primers used in the study are listed in Table S4. The quality of the amplified fragments was controlled by the melting curve. Transcipt levels were normalized with *GAPDH* and relative mRNA levels in experimental samples were normalized to controls.

### ChIP-PCR

Cells were grown to 80% confluence and cells were then crosslinked with 1% formaldehyde and processed. The crosslinking, immunoprecipitation, washing, elution, reverse crosslinking, and proteinase K treatment were performed according to the manufacturer’s directions described in the Magna ChIP G Chromatin Immunoprecipitation Kit from Millipore. Purified immunoprecipitated DNA was used for quantitative RT-PCR.

### Luciferase reporter assay

HEK293T cells were seeded in 12-well plates 24 h before transfection. The following day, various plasmids together with reporter plasmid and *Renilla*-luciferase control vector was co-transfected. Cells were collected 48 h post-transfection and assayed for luciferase activity by using a Varioskan Flash spectral scanning multimode reader (Thermo Scientific).

### In vitro deacetylation assay

Constructs encoding FLAG-tagged PRRX1 and PRRX1^K160R^ were separately transfected in HEK293T cells. The lysates were immunoprecipitated by anti-FLAG^®^ M2 Agarose (Sigma-Aldrich). The bound proteins were eluted by competition with a large excess of free FLAG peptides. Purified proteins were incubated with 1 µg GST-SIRT1 protein in the deacetylation buffer (50 mM Tris–HCl [pH 8.0], 0.8 mM MgCl_2_, and/or 1 mM NAD+. Meanwhile, 10 mM NAM) at 30 °C for 15 min with constant agitation. Meanwhile, 10 mM NAM was added in one reaction to inhibit SIRT1 deacetylase activity. The reactions were then stopped and the acetylation level was determined by western blotting with anti-acetyl lysine antibody.

### Tumorigenesis and metastasis assay

10^5^ 4T1 cells were subcutaneously injected into the inguinal mammary gland. Tumor volume (mm^3^) was determined by measuring length (*l*) and width (*w*) and calculating volumes (*V* = lw^2^/2). Control and mutant BT-549 cells were propagated as monolayers and trypsinized. For tail injection, cells were resuspended in PBS at a concentration of 5 × 10^6^ cells/ml. 100 μl cell suspension was injected into tail veins. At the endpoint, all of the mice were sacrificed and the tissues were removed, fixed in 4% Paraformaldehyde (PFA), paraffin-embedded and sectioned. The TUNEL working procedure was carried out following the producer’s directions (Roche).

### Immunohistochemistry

Paraffin-embedded sections of PFA-fixed tumors were stained with anti-E-Cadherin monoclonal antibody. HRP-conjugated anti-mouse secondary antibody was added and detected according to the manufacturer’s protocol (Daco).

### Human breast cancer array

The human tissue array (catalog# BR2082a and BR2082b) was bought from Cybrdi Shanxi ChaoYing Biotechnology, China. All the pathologic diagnosis was supplied in the manufacturer’s instructions of the product. Tissue staining was classified as negative, low, medium, and high according to the staining intensity and the percentage of positive cells. A χ2 test was used to evaluate where there is a significant association between SIRT1 and Prrx1 or KLF4 expression.

### Statistical analyses

Data were analyzed using Prism 5.0 (GraphPad Software). The correlations were determined by a Pearson’s coefficient of correlation (r). Statistical comparisons between two groups were accessed by two-tailed student’s *t*-tests. Error bar represents standard error of the mean (SEM). The use of statistical tests was chosen according to the nature of the data. Statistical significance was defined as *P* values of<0.05.

## Electronic supplementary material

Supplementary Information

Supplementary Figure S1

Supplementary Figure S2

Supplementary Figure S3

Supplementary Figure S4

Supplementary Figure S5

Supplementary Figure S6

Supplementary Figure S7

Supplementary Figure S8

Supplementary Figure S9

Supplementary Figure S10

Supplementary Figure S11

Supplementary Table S2

Supplementary Table S3
