# Supplementary material for: A SIRT1-centered circuitry regulates breast cancer stemness and metastasis
Source: Oncogene. 2018 Jul 23;37(49):6299–315. doi: 10.1038/s41388-018-0370-5 (PMC6283862; doi:10.1038/s41388-018-0370-5)
Supplement: Supplementary file 1 — Supplementary Information [file 41388_2018_370_MOESM1_ESM.docx]

**Supplementary Information**

**Supplementary Table S1. Correlation between 4 stemness factors, CSC markers and age-related genes in breast cancer cell lines**

| **Correlation between 4 factors and age-related genes in breast cancer cells** | | | | | |
| --- | --- | --- | --- | --- | --- |
| **KLF4** | **r (Pearson )** | ***P* value** | **KLF4** | **r (Pearson )** | ***P* value** |
| E2F1 | -0.3762 | 0.0051 | HDAC1 | -0.2577 | 0.06 |
| STAT5B | -0.284 | 0.0374 | FGFR1 | -0.3342 | 0.0136 |
| PARP1 | -0.2763 | 0.0432 | CCNA2 | -0.2694 | 0.0488 |
| PIN1 | -0.3588 | 0.0078 | HMGB2 | -0.344 | 0.0108 |
| BLM | -0.3671 | 0.0064 | LMNB1 | -0.3029 | 0.026 |
| POLG | -0.298 | 0.0286 | BUB1B | -0.4345 | 0.001 |
| HSP90AA1 | -0.3124 | 0.0214 | SIN3A | -0.3247 | 0.0166 |
| TOP2A | -0.3995 | 0.0028 | H2AFX | -0.2897 | 0.0336 |
| ERCC3 | -0.3216 | 0.0178 | ATR | -0.3235 | 0.017 |
| FEN1 | -0.3962 | 0.003 | TP53BP1 | -0.3092 | 0.0229 |
| FOXM1 | -0.3359 | 0.013 | HTRA2 | -0.3407 | 0.0117 |
| SIRT1 | -0.3038 | 0.0256 | NUDT1 | -0.4737 | 0.0003 |
| **POU1F1** | **r (Pearson )** | ***P* value** | **POU1F1** | **r (Pearson )** | ***P* value** |
| STAT3 | -0.29 | 0.0334 | MAPK14 | -0.2928 | 0.0317 |
| INSR | -0.3588 | 0.0077 | TPP2 | -0.3098 | 0.0226 |
| BRCA1 | -0.3013 | 0.0268 | GRN | -0.3107 | 0.0222 |
| TERF2 | -0.3328 | 0.0139 |  | - |  |
| **SOX2** | **r (Pearson )** | ***P* value** | **SOX2** | **r (Pearson )** | ***P* value** |
| SHC1 | -0.3137 | 0.0209 | IL6 | -0.2902 | 0.0332 |
| ATM | -0.3878 | 0.0038 | GPX1 | -0.4722 | 0.0004 |
| PLAU | -0.3493 | 0.0096 | JUN | -0.2957 | 0.03 |
| LMNA | -0.4858 | 0.0002 | CTGF | -0.3383 | 0.0124 |
| NFKB2 | -0.2897 | 0.0336 | JAK2 | -0.4398 | 0.0008 |
| STAT3 | -0.3803 | 0.0046 | LEPR | -0.3058 | 0.0245 |
| STAT5A | -0.382 | 0.0044 | DBN1 | -0.2977 | 0.0288 |
| IL7R | -0.3352 | 0.0132 | ATR | -0.3379 | 0.0125 |
| MYC | -0.407 | 0.0023 | GCLC | -0.2875 | 0.035 |
| EGFR | -0.4728 | 0.0003 | GCLM | -0.4996 | 0.0001 |
| HIF1A | -0.2939 | 0.031 | PAPPA | -0.2704 | 0.048 |
| XRCC5 | -0.3131 | 0.0212 | EIF5A2 | -0.2801 | 0.0402 |
| SOD2 | -0.3936 | 0.0032 |  |  |  |
| **NANOG** | **r (Pearson )** | ***P* value** | **NANOG** | **r (Pearson )** | ***P* value** |
| TXN | -0.2911 | 0.0327 | XRCC5 | -0.3661 | 0.0065 |
| STAT5A | -0.2853 | 0.0365 | PCNA | -0.3197 | 0.0185 |
| IL7R | -0.3313 | 0.0144 | FEN1 | -0.3926 | 0.0033 |
| EGFR | -0.2936 | 0.0312 | TERF2 | -0.3294 | 0.015 |
| BRCA1 | -0.3446 | 0.0107 | HDAC1 | -0.401 | 0.0027 |
| HIF1A | -0.3393 | 0.0121 | GPX1 | -0.2948 | 0.0305 |
| RPA1 | -0.3279 | 0.0155 | MAPK8 | -0.3232 | 0.0171 |
| BLM | -0.3391 | 0.0121 | IL7 | -0.3231 | 0.0172 |
| IGFBP3 | -0.3598 | 0.0075 | JUN | -0.2881 | 0.0347 |
| NR3C1 | -0.3021 | 0.0264 | HMGB2 | -0.2706 | 0.0478 |
| ABL1 | -0.3287 | 0.0152 | DBN1 | -0.3331 | 0.0139 |
| TOP2A | -0.3304 | 0.0147 | NOG | -0.289 | 0.0341 |
| TGFB1 | -0.4223 | 0.0015 | CDC42 | -0.3599 | 0.0075 |
| PML | -0.4214 | 0.0015 | NFE2L2 | -0.3045 | 0.0252 |
| HELLS | -0.3508 | 0.0093 | EIF5A2 | -0.371 | 0.0057 |
| **Correlation between CSC markers and age-related genes in breast cancer cells** | | | | | |
| **ALDH1A1** | **r (Pearson )** | ***P* value** | **ALDH1A1** | **r (Pearson )** | ***P* value** |
| WRN | -0.3168 | 0.0196 | MSRA | -0.2825 | 0.0385 |
| HRAS | -0.2747 | 0.0444 | TOP3B | -0.3119 | 0.0217 |
| BLM | -0.2926 | 0.0318 | SIRT1 | -0.2283 | 0.0485 |
| POLG | -0.2771 | 0.0425 | HSPD1 | -0.2843 | 0.0372 |
| ABL1 | -0.3089 | 0.023 | BMI1 | -0.3216 | 0.0177 |
| HELLS | -0.3104 | 0.0224 | HSPA8 | -0.3126 | 0.0214 |
| EEF2 | -0.488 | 0.0002 | SIN3A | -0.2806 | 0.0399 |
| TERF1 | -0.4236 | 0.0014 | SIRT6 | -0.2957 | 0.0299 |
| PRKDC | -0.4297 | 0.0012 | SOCS2 | -0.3884 | 0.0037 |
| XRCC5 | -0.2804 | 0.04 |  |  |  |
| **ALDH1A3** | **r (Pearson )** | ***P* value** | **ALDH1A3** | **r (Pearson )** | ***P* value** |
| GHR | -0.3483 | 0.0098 | BMI1 | -0.2965 | 0.0295 |
| AKT1 | -0.2696 | 0.0486 | HOXC4 | -0.2723 | 0.0464 |
| AR | -0.3736 | 0.0054 | ESR1 | -0.4252 | 0.0014 |
| LRP2 | -0.3931 | 0.0033 | CLU | -0.4233 | 0.0014 |
| HSPA9 | -0.343 | 0.0111 | PPP1CA | -0.2875 | 0.0351 |
| FGFR1 | -0.4434 | 0.0008 | UCP3 | -0.298 | 0.0286 |
| SIRT1 | -0.2881 | 0.0347 | IKBKB | -0.2868 | 0.0355 |
| **CD44** | **r (Pearson )** | ***P* value** | **CD44** | **r (Pearson )** | ***P* value** |
| GHR | -0.4164 | 0.0017 | CLU | -0.4768 | 0.0003 |
| GHRH | -0.2848 | 0.0368 | GHRHR | -0.3629 | 0.007 |
| PROP1 | -0.2998 | 0.0277 | PPP1CA | -0.3768 | 0.005 |
| EPOR | -0.3701 | 0.0059 | UCP3 | -0.3967 | 0.003 |
| SST | -0.2767 | 0.0428 | UCP2 | -0.5407 | < 0.0001 |
| PRKCD | -0.4325 | 0.0011 | STUB1 | -0.3963 | 0.003 |
| RET | -0.3722 | 0.0056 | PPM1D | -0.358 | 0.0079 |
| NFKB1 | -0.3695 | 0.006 | ARHGAP1 | -0.3925 | 0.0033 |
| PDPK1 | -0.472 | 0.0003 | ADCY5 | -0.4521 | 0.0006 |
| CEBPA | -0.5347 | < 0.0001 | GPX4 | -0.3283 | 0.0154 |
| AR | -0.5779 | < 0.0001 | FGF23 | -0.3909 | 0.0035 |
| GRB2 | -0.328 | 0.0155 | NCOR2 | -0.284 | 0.0374 |
| COQ7 | -0.3212 | 0.0179 | IGFBP2 | -0.2765 | 0.043 |
| SNCG | -0.3081 | 0.0234 | PYCR1 | -0.4783 | 0.0003 |
| FOXO4 | -0.4723 | 0.0003 | SIRT7 | -0.2604 | 0.0385 |
| BSCL2 | -0.3579 | 0.0079 | C1QA | -0.2876 | 0.0349 |
| TFAP2A | -0.3074 | 0.0238 | DGAT1 | -0.3368 | 0.0128 |
| MAPT | -0.3374 | 0.0126 | FGF21 | -0.282 | 0.0388 |
| ESR1 | -0.3529 | 0.0089 | IKBKB | -0.3025 | 0.0262 |
| LEP | -0.3238 | 0.0169 | TRPV1 | -0.4013 | 0.0026 |

**Supplementary Table S2. Correlation between stemness-related factors in OCT4-SOX2-NANOG-KLF4 core circuitry, phenotypic and functional markers of CSCs and SIRT1 in breast cancer cells**

**Supplementary Table S3. GenAge Human Genes list and the key KEGG pathways of selected age-related and stemness-associated genes**

**Supplementary Table S4. Oligonucleotides used in experiments**

| **Name** | **Sequence (5’-3’)** | **Purposes** |
| --- | --- | --- |
| Kozak-Cre-NheI-F | AGCGCTAGCCACCATGTCCAATTTACTGACCG | pCDH-Cre-GFP |
| Cre-NotI-R | AGAGCGGCCGCTAATCGCCATCTTCCAGCA | pCDH-Cre-GFP |
| Kozak-PRRX1-NheI-F | AGCGCTAGCCACCATGACCTCCAGCTACGGGC | pcDNA-PRRX1-  Myc/His |
| Kozak-3FLAG-NheI-F | TACGCTAGCCACCATGGACTACAAAGACCATGACGGTGATTACAAGGATGACGACGATAA | pcDNA-3FLAG-PRRX1-Myc/His |
| FLAG-PRRX1-F | CAAGGATGACGACGATAAGGATTACAAGGATGACGCGATAAGACCTCCAGCTACGGGC | pcDNA-3FLAG-PRRX1-Myc/His |
| PRRX1-XhoI-F | CTCAGATCTCGAGCGACCTCCAGCTACGGGCAC | pDsRed-PRRX1 |
| PRRX1-NdeI-F | AGCCATATGACCTCCAGCTACGGGC | pET28a-PRRX1 |
| 6His-XhoI-R | TAGCTCGAGTCAATGATGATGATGATGATG | pET28a-PRRX1 |
| Prrx1a-HindIII-R | AGAAAGCTTTCCCTGTACGGAGAGGCTGTCC | PRRX1a constructs |
| Prrx1b-HindIII-R | AGAAAGCTTTCGAATCCGTTATGAAGCCCCTC | PRRX1b constructs |
| 160KR-R | GAGAAGCGTTCCTATTGGCCAGCATGGCTCG | PRRX1K160R |
| 160KR-F | CTGGCCAATAGGAACGCTTCTCTCCTCAAGTC | PRRX1K160R |
| 160KQ-R | GAGAAGCGTTTTGATTGGCCAGCATGGCTCG | PRRX1K160Q |
| 160KQ-F | CTGGCCAATCAAAACGCTTCTCTCCTCAG | PRRX1K160Q |
| 31/32KR-R | ACGGAGAAGTTCCTTCTCGCCTGCAGGGTGTCG | PRRX1K31/32R |
| 31/32KR-F | CCCTGCAGGCGAGAAGGAACTTCTCCGTCAGTC | PRRX1K31/32R |
| 4KR-R | GTTGTCCTGTTTCTCCGCTGCCTTCTCCTCCTCCTCTCCT CAGAGTTCAACTG | PRRX1K89/90/91/93R |
| 4KR-F | CAGTTGAACTCTGAGGAGAGGAGGAGGAGAAGGCAGCGG AGAAACAGGA CAAC | PRRX1K89/90/91/93R |
| 148KR-R | CGCTCATTCCTGCGGAACCTGGCTCTTCGGTTCTGAA | PRRX1K148R |
| 148KR-F | TTCAGAACCGAAGAGCCAGGTTCCGCAGGAATGAGCG | PRRX1K148R |
| 166KR-R | ATAAAAACGCTTCTCTCCTCAGGTCCTACTCAGGAGACGTG | PRRX1K166R |
| 166KR-F | CACGTCTCCTGAGTAGGACCTGAGGAGAGAAGCGTTTTTAT | PRRX1K166R |
| SIRT1g-F | CACCGATAGCAAGCGGTTCATCAGC | pX459-SIRT1g |
| SIRT1g-R | AAACGCTGATGAACCGCTTGCTATC | pX459-SIRT1g |
| SIRT1 KO-F | CCCATCATTGACAGTTGCT | Sequencing |
| SIRT1 KO-R | TGAGGGAAGACCCAATAAC | Sequencing |
| PRRX1g-F | CACCGCTCCAGAACGTGCCCGTAGC | pX459-PRRX1g |
| PRRX1g-R | AAACGCTACGGGCACGTTCTGGAGC | pX459-PRRX1g |
| PRRX1 KO-F | TTCCCCACTCGGCTCCTCTC | Sequencing |
| PRRX1 KO-R | TCACTCACTGTCCTGCTGCGG | Sequencing |
| KLF4P-F-NheI | GCGCTAGCTCTCGCTATTTAAAGTATCAA | pGL3-KLF4 |
| KLF4P-R-HindIII | TATAAGCTTCCTGCGAGCAAGGCAGGGAGC | pGL3-KLF4 |
| KLF4P-mut-F | GCGGATAGCTCTCGCTATTTAAAGTATCAA | pGL3-KLF4 mut |
| KLF4P-mut-R | AAACCCCAGGCCGGCCGAGATCCTTCTTCTTTG | pGL3-KLF4 mut |
| ALDH1A1P-F-NheI | ACTGCTAGCTGGCCCGTGCACCAAATCTGG | pGL3-ALDH1A1 |
| ALDH1A1P-R-HindIII | GCGAAGCTTGGAACACAGGTGACTGGCTCA | pGL3-ALDH1A1 |
| ALDH1A3P-F-NheI | GTCTGTCCTGCTAGCGGGGATC | pGL3-ALDH1A3 |
| ALDH1A3P-R-HindIII | ACTAAGCTTGGACACTGCGCAGCCCGCTCC | pGL3-ALDH1A3 |
| shSIRT1-F | AACGTATTGCTGAACAGATGGAACTCGAGTTCCATCTGTTCAGCAATACTTTTTC | shSIRT1 |
| shSIRT1-R | TCGAGGAAAAAGTATTGCTGAACAGATGGAACTCGAGTTCCATCTGTTCAGCAATAC | shSIRT1 |
| shKLF4-F | AACGCCTTACACATGAAGAGGCATCTCGAGATGCCTCTTCATGTGTAAGGCTTTTTC | shKLF4 |
| shKLF4-R | TCGAGGAAAAAGCCTTACACATGAAGAGGCATCTCGAGATGCCTCTTCATGTGTAAGGC | shKLF4 |
| mGAPDH-R | TGTAGACCATGTAGTTGAGGTCA | qRT-PCR |
| mCDH1-F | GCACTCTTCTCCTGGTCCTG | qRT-PCR |
| mCDH1-R | TATGAGGCTGTGGGTTCCTC | qRT-PCR |
| mCDH2-F | CATTATCAACCCCATCTCAGG | qRT-PCR |
| mCDH2-R | TGCATGTGCTCTCAAGTGAA | qRT-PCR |
| mFN1-F | GGAATGGACCTGCAAACCTAT | qRT-PCR |
| mFN1-R | CATCATCCAGCCTTGGTAGG | qRT-PCR |
| PRRX1-F | TTACCCGGATGCTTTTGTTC | qRT-PCR |
| PRRX1a-R | AAGTAGCCATGGCGCTGTA | qRT-PCR |
| PRRX1b-R | GCCCCTCGTGTAAACAACAT | qRT-PCR |
| mSNAIl-F | CACACGCTGCCTTGTGTCT | qRT-PCR |
| mSNAIl-R | GGTCAGCAAAAGCACGGTT | qRT-PCR |
| mSNAI2-F | CTCACCTCGGGAGCATACAGC | qRT-PCR |
| mSNAIL2-R | TGAAGTGTCAGAGGAAGGCGGG | qRT-PCR |
| mTWIST-F | CGGGTCATGGCTAACGTG | qRT-PCR |
| mTWIST-R | CAGCTTGCCATCTTGGAGTC | qRT-PCR |
| mVIM-F | CGTCCACACGCACCTACAG | qRT-PCR |
| mVIM-R | GGGGGATGAGGAATAGAGGCT | qRT-PCR |
| mZEB1-F | TTCAAACCCATAGTGGTTGCT | qRT-PCR |
| mZEB1-R | TGGGAGATACCAAACCAACTG | qRT-PCR |
| mZEB2-F | ATTGCACATCAGACTTTGAGGAA | qRT-PCR |
| mZEB2-R | ATAATGGCCGTGTCGCTTCG | qRT-PCR |
| mCD133-F | GAAAAGTTGCTCTGCGAACC | qRT-PCR |
| mCD133-R | TCTCAAGCTGAAAAGCAGCA | qRT-PCR |
| mCD24a-F | ACCCACGCAGATTTACTGCAA | qRT-PCR |
| mCD24a-R | CCCCTCTGGTGGTAGCGTTA | qRT-PCR |
| mCD44-F | TCTGCCATCTAGCACTAAGAGC | qRT-PCR |
| mCD44-R | GTCTGGGTATTGAAAGGTGTAGC | qRT-PCR |
| mKLF4-F | GCGAACTCACACAGGCGAGAAACC | qRT-PCR |
| mKLF4-R | TCGCTTCCTCTTCCTCCGACACA | qRT-PCR |
| mMYC-F | TGACCTAACTCGAGGAGGAGCTGGAATC | qRT-PCR |
| mMYC-R | AAGTTTGAGGCAGTTAAAATTATGGCTGAAGC | qRT-PCR |
| mNANOG-F | TTGCTTACAAGGGTCTGCTACT | qRT-PCR |
| mNANOG-R | ACTGGTAGAAGAATCAGGGCT | qRT-PCR |
| mNOTCH1-F | GCCGCAAGAGGCTTGAGAT | qRT-PCR |
| mNOTCH1-R | GGAGTCCTGGCATCGTTGG | qRT-PCR |
| mOCT4-F | AGTTGGCGTGGAGACTTTGC | qRT-PCR |
| mOCT4-R | CAGGGCTTTCATGTCCTGG | qRT-PCR |
| mREX1-F | CGTGTAACATACACCATCCG | qRT-PCR |
| mREX1-R | GAAATCCTCTTCCAGAATGG | qRT-PCR |
| mSSEA1-F | GGAGGGAGCAGTGACGCTAAC | qRT-PCR |
| mSSEA1-R | GTATGGGAGGGCGATTCGA | qRT-PCR |
| mSOX9-F | ACGGCTCCAGCAAGAACAAG | qRT-PCR |
| mSOX9-R | TTGTGCAGATGCGGGTACTG | qRT-PCR |
| mSO2-F | GCGGAGTGGAAACTTTTGTCC | qRT-PCR |
| mSOX2-R | CGGGAAGCGTGTACTTATCCTT | qRT-PCR |
| hCDH1-F | CCCGGGACAACGTTTATTAC | qRT-PCR |
| hCDH1-R | GCTGGCTCAAGTCAAAGTCC | qRT-PCR |
| hCDH2-F | TGCACAGATGTGGACAGGAT | qRT-PCR |
| hCDH2-R | CCACAAACATCAGCACAAGG | qRT-PCR |
| hFN-F | GAACTATGATGCCGACCAGAA | qRT-PCR |
| hFN-R | GGTTGTGCAGATTTCCTCGT | qRT-PCR |
| hVIM-F | AAAGTGTGGCTGCCAAGAAC | qRT-PCR |
| hVIM-R | AGCCTCAGAGAGGTCAGCAA | qRT-PCR |
| hSIRT1-F | TACCGAGATAACCTTCTGTTCG | qRT-PCR |
| hSIRT1-R | GTTCGAGGATCTGTGCCAAT | qRT-PCR |
| hPRRX1-F | GCACAGGCGGATGAGAAC | qRT-PCR |
| hPRRX1-R | TCTTCTGAGTTCAGCTGGTCAT | qRT-PCR |
| hPRRX1-F | ACTTGGCTCTTCGGTTCTGA | qRT-PCR |
| hPRRX1-R | ACTTGGCTCTTCGGTTCTGA | qRT-PCR |
| hTWIST1-F | AGCTACGCCTTCTCGGTCT | qRT-PCR |
| hTWIST1-R | CCTTCTCTGGAAACAATGACATC | qRT-PCR |
| hSNAI1-F | GCTGCAGGACTCTAATCCAGA | qRT-PCR |
| hSNAI1-R | ATCTCCGGAGGTGGGATG | qRT-PCR |
| hSNAI2-F | ACAGCGAACTGGACACACAT | qRT-PCR |
| hSNAI2-R | GATGGGGCTGTATGCTCCT | qRT-PCR |
| hZEB1-F | TTTTTCCTGAGGCACCTGAA | qRT-PCR |
| hZEB1-R | AAAATGCATCTGGTGTTCCAT | qRT-PCR |
| hZEB2-F | AAGCCAGGGACAGATCAGC | qRT-PCR |
| hZEB2-R | GCCACACTCTGTGCATTTGA | qRT-PCR |
| hNANOG-F | ATGCCTCACACGGAGACTGT | qRT-PCR |
| hNANOG-R | AGGGCTGTCCTGAATAAGCA | qRT-PCR |
| hMYC-F | CACCAGCAGCGACTCTGA | qRT-PCR |
| hMYC-R | GATCCAGACTCTGACCTTTTGC | qRT-PCR |
| hOCT4-F | GTGCCTGCCCTTCTAGGAAT | qRT-PCR |
| hOCT4-R | GGCACAAACTCCAGGTTTTCT | qRT-PCR |
| hKLF9-F | CTCCGAAAAGAGGCACAAGT | qRT-PCR |
| hKLF9-R | CGGGAGAACTTTTTAAGGCAGT | qRT-PCR |
| hSOX2-F | TATTTGAATCAGTCTGCCGAG | qRT-PCR |
| hSOX2-R | ATGTACCTGTTATAAGGATGATATTAGT | qRT-PCR |
| hSOX9-F | AGGTGCTCAAAGGCTACGAC | qRT-PCR |
| hSOX9-R | GTAATCCGGGTGGTCCTTCT | qRT-PCR |
| hKLF4-F | GAAATTCGCCCGCTCCGATGA | qRT-PCR |
| hKLF4-R | CTGTGTGTTTGCGGTAGTGCC | qRT-PCR |
| hKLF4-F | TCTCAAGGCACACCTGCGAA | qRT-PCR |
| hKLF4-R | TAGTGCCTGGTCAGTTCATC | qRT-PCR |
| KLF4 P1-F | GTGAGTACGGCCCTGGTCGCGCAGC | qRT-PCR |
| KLF4 P1-R | GCAGTGGTGTCGGCGGCGGCGGCGG | qRT-PCR |
| KLF4 P2-F | CGCTGAGCGACGAGAGCGGACTCCT | qRT-PCR |
| KLF4 P2-R | TGGAGAAGAGCGCGATTATCCGCGT | qRT-PCR |
| KLF4 P3-F | TGCCTCTTTCCGCCTGTT | qRT-PCR |
| KLF4 P3-R | GACCTCGCACGGTTCCTC | qRT-PCR |
| KLF4 P4-F | TGATCACAAACCAAGGGG | qRT-PCR |
| KLF4 P4-R | GTGACCATGTGCCAGGAA | qRT-PCR |
| KLF4 P5-F | GGACGCTGCTGAGTGGAAGAG | qRT-PCR |
| KLF4 P5-R | TCCTCACCCCTCCCTGCTC | qRT-PCR |
| ALDH1A1-F | CTGCTGGCGACAATGGAGT | qRT-PCR |
| ALDH1A1-R | GTCAGCCCAACCTGCACAG | qRT-PCR |
| ALDH1A3-F | TCTCGACAAAGCCCTGAAGT | qRT-PCR |
| ALDH1A3-R | TATTCGGCCAAAGCGTATTC | qRT-PCR |
| hGAPDH-F | TTCGACAGTCAGCCGCATCTTCTT | qRT-PCR |
| hGAPDH-R | TGTCATCATATTTGGCAGGTT | qRT-PCR |
| hGAPDH-F | TTGGTATCGTGGAAGGACTCA | qRT-PCR |
| hGAPDH-R | TGTCATCATATTTGGCAGGTT | qRT-PCR |

**Supplementary Table S5. List of antibodies used in this study**

| **Antibody** | **Company** | **Purposes** |
| --- | --- | --- |
| Rb α acetyl lysine | Abcam | WB |
| Rb α acetyl lysine | EMD Millipore | WB |
| Ms α ß-Actin | Sigma-Aldrich | WB |
| Ms α ALDH1 | BD Biosciences | IF and WB |
| Rb α ALDH1A1 | Abcam | WB |
| Ms α E-Cadherin | BD Biosciences | IF |
| Ms α E-Cadherin | BD Biosciences | IF |
| Rb α E-Cadherin | Cell Signaling Technology | WB |
| Ms α N-Cadherin | BD Biosciences | WB |
| Rb α ß-Catenin | Cell Signaling Technology | WB |
| Rb α CD24 | Abcam | IF |
| Ms α CD44 | BD Biosciences | IF |
| Ms α CK5 | Abcam | IF |
| Rb α CK8 | Abcam | IF |
| Rb α Cre | EMD Millipore | WB |
| Ms α FLAG | Sigma-Aldrich | IP and WB |
| Rb α Fibronectin | Abcam | WB |
| Ms α GAPDH | Abcam | WB |
| Ms α GST | Cell Signaling Technology | WB |
| Ms α HA | Santa Cruz | IP |
| Rb α KLF4 | Abcam | WB and IHC |
| Ms α c-Myc | Sigma-Aldrich | WB |
| Ms α Nanog | Santa Cruz | IF |
| Goat α Prrx1 | Abnova | IF and WB |
| Rb α Prrx1 | Abcam | IP, WB and IHC |
| Rb α Snail1 | Cell Signaling Technology | WB |
| Ms α SSEA1 | Abcam | IF |
| Ms α SIRT1 | Santa Cruz | WB |
| Rb α SIRT1 | Abcam | IF, WB and IHC |
| Rb α SIRT1 | Santa Cruz | IP and WB |
| Rb α Sox2 | Cell Signaling Technology | IF |
| Rb α Sox9 | EMD Millipore | IF |
| Ms α ß-Tubulin | Sigma-Aldrich | WB |
| Ms α Twist1 | Abcam | WB |
| Ms α Ubiquitin | Cell Signaling Technology | WB |
| Rb α Vimentin | Cell Signaling Technology | WB |
| Rb α Zeb1 | Bethyl Laboratories | WB |
| Rb α Zeb2 | Santa Cruz | WB |

Abbreviations: Western blotting (WB), Immunoﬂuorescence staining (IF), Immunoprecipitation assay (IP), Immunohistochemistry assay (IHC) and flow cytometry (FACS).

**Supplementary Figures**

**Supplementary Figure S1. Differential roles of sirtuins in regulating CSCs**

(**A**) Representative images showing mammospheres derived from BT549 cells stably transfected with pIRES-EGFP (empty vector) or pSIRT7-IRES-EGFP (*SIRT7* OE). Noted that *SIRT7* OE significantly increased the size and number of mammospheres.

(**B**) GSEA showing EMT-induced CSC signature is increased in *SIRT7*-depleted BT549 cells. Normalized enrichment score (NES) are shown in the plot.

(**C**) Heat map depicting differential expression of genes in ALDH^+^ breast cancer cells compared with ALDH^-^ cells (GSE52327). Red and green indicate high and low mRNA level respectively.
(**D**) Heat map depicting differential expression of genes in CD24^-^/CD44^+^ breast cancer cells compared with non-CD44^-^/CD24^+^ cells (GSE52262). Red and green indicate high and low mRNA level respectively.

(**E**) Fold changes of indicated genes in CD24^−^CD44^+^ versus others and ALDH^+^ versus ALDH^−^ cells isolated from breast tumors.

**Supplementary Figure S2. Correlation of genes with RFS and DMFS in patients with breast cancer.**

(**A**) Kaplan-Meier plots showing correlation between indicated genes and relapse-free survival (RFS) and distant metastasis free survival (DMFS) in breast cancers.

(**B**) Kaplan-Meier plots showing RFS with or without systemic treatments and DMFS of breast cancers based on *SIRT1* level.

**Supplementary Figure S3. Silencing *Sirt1* induces CSCs in murine breast cancer cells.**

(**A**) Representative images showing the level of *Sirt1* in *Sirt1* KD 4T1 cells.

(B) Representative images showing mammospheres derived from Control and *Sirt1* KD 4T1 cells. Scale bar, 100 µm.

(**C**) Quantification of mammosphere-forming efficiency. Noted that *Sirt1* KD increased the size and number of mammospheres. Data represent mean ± SEM of 3 independent experiments. ***P* < 0.01

(**D**) Quantitative RT-PCR analyses of mRNA level of stemness-related genes in Control and *Sirt1* KD 4T1 cells.

(**E**) Quantitative RT-PCR analyses of mRNA levels of EMT inducers in Control and *Sirt1* KD 4T1 cells.

**Supplementary Figure S4. *SIRT1* deficiency induces ALDH+ CSC properties via activating KLF4.**

(**A**) Heat map depicting differential expression of stemness-related genes in *SIRT1 KO* (*SKO*) BT549 compared with control cells. Red and green indicate high and low mRNA levels respectively. Noted that *ALDH1A1* and *KLF4* were significantly upregulated in *SKO* cells. Significance was determined by a two-tailed, unpaired Student’s *t*-test.

(**B**) Correlation between mRNA levels of *KLF4* and *ALDH1A1/ALDH1A3* in 683 breast cancer patients (based on Pearson’s correlation coefficient).

(**C**) Predicted KLF4 binding sites on human *ALDH1A1* and *ALDH1A3* promoter.
(**D**) Luciferase assays in HEK293T cells co-transfected with empty vector (EV) or HA-KLF4 plasmid together with *ALDH1A1* or *ALDH1A3* promoter reporter plasmids. (n = 3; * *P* < 0.05, *t*-test).

(**E**) Proliferation curves of indicated cells. Data represent mean ± SEM of 3 independent experiments. (n = 3; * *P* < 0.05, ** *P* < 0.01, *t*-test).

**Supplementary Figure S5. KLF4 is less likely regulated by SIRT1 via chromatin remodeling.**

(**A**) H3K9ac levels in Control and *SIRT1* KO BT549 cells.

(**B**) ChIP-PCR assay using anti SIRT1 antibody or control IgG in BT549 cells.

(**C**) H3K9ac-ChIP enrichment at *KLF4* promoter in Control and *SIRT1* KO BT549 cells (n = 3).

**SupplementaryFigure S6. Quantitative RT-PCR analyses of mRNA levels of EMT inducers.**

**Supplementary Figure S7. KLF4 is Prrx1’s direct target**

(**A**) Predicted PRRX1 binding sites on human *KLF4* promoter.

(**B**) Representative images showing functional domains and difference of Prrx1a and Prrx1b.

(**C**) Quantitative RT-PCR analysis of *PRRX1A* and *PRRX1B* mRNA levels in BT549 cells. Values are presented as relative to *PRRX1A* mRNA transcripts. (n = 3; * *P* < 0.05, *t*-test).

**Supplementary Figure S8. Co-localization of GFP-SIRT1 and Ds-Red-PRRX1.**

Scale bar, 5 µm.

**Supplementary Figure S9. SIRT1 deacetylates PRRX1 and prevents its degradation**

(**A**) Representative immunoblots showing acetyl lysine levels of Myc-PRRX1 treated with indicated HDAC inhibitors.

(**B**) Representative immunoblots showing acetyl lysine levels of FLAG-PRRX1 treated with si-*SIRT1* or Scramble.

(**C**) Protein levels of endogenous Prrx1 in MEF in presence or absence of CHX and/or MG132.

(**D**) Protein levels of Myc-PRRX1 in HEK293 cell treated with HDAC inhibitors.

**Supplementary Figure S10. Suppressing KLF4 sensitizes SIRT1 deficiency-induced chemoresistance**

(**A**) SKO BT549 tumor cells (5 ×10^6^ cells per mouse) were injected intravenously. Mice were treated vehicle, PTX (25 mg kg-1 body weight) and PTX plus 200 μg KEN per mouse per week (n=8 per group). Cumulative survival of mice with indicated treatments. (Kaplan-Meier, with log-rand test).

(**B**-**C**) Representative H&E-stained sections of lung (**B**) and quantification of the percentages of metastatic areas (**C**) collected from the different treatment groups of mice. Black arrows indicate lung metastasis. Data are represented as mean ± SEM (n= 4 independent experiments). Scale bar, 1 mm.

**Supplementary Figure S11. Summary of clinicopathologic parameters and representative IHC images of human breast cancer arrays.**

(**A**-**C**) Association between SIRT1, PRRX1 or KLF4 levels and clinicopathologic parameters of human breast cancer arrays.

(**D**) Representative images showing a typical nuclear-SIRT1^Low^-PRRX1^Low^-KLF4^High^ IHC straining in tumor issues of patient 27#. Scale bar, 100 µm.
