# Supplementary figures and images for: A SIRT1-centered circuitry regulates breast cancer stemness and metastasis
Source: Oncogene. 2018 Jul 23;37(49):6299–315. doi: 10.1038/s41388-018-0370-5 (PMC6283862; doi:10.1038/s41388-018-0370-5)

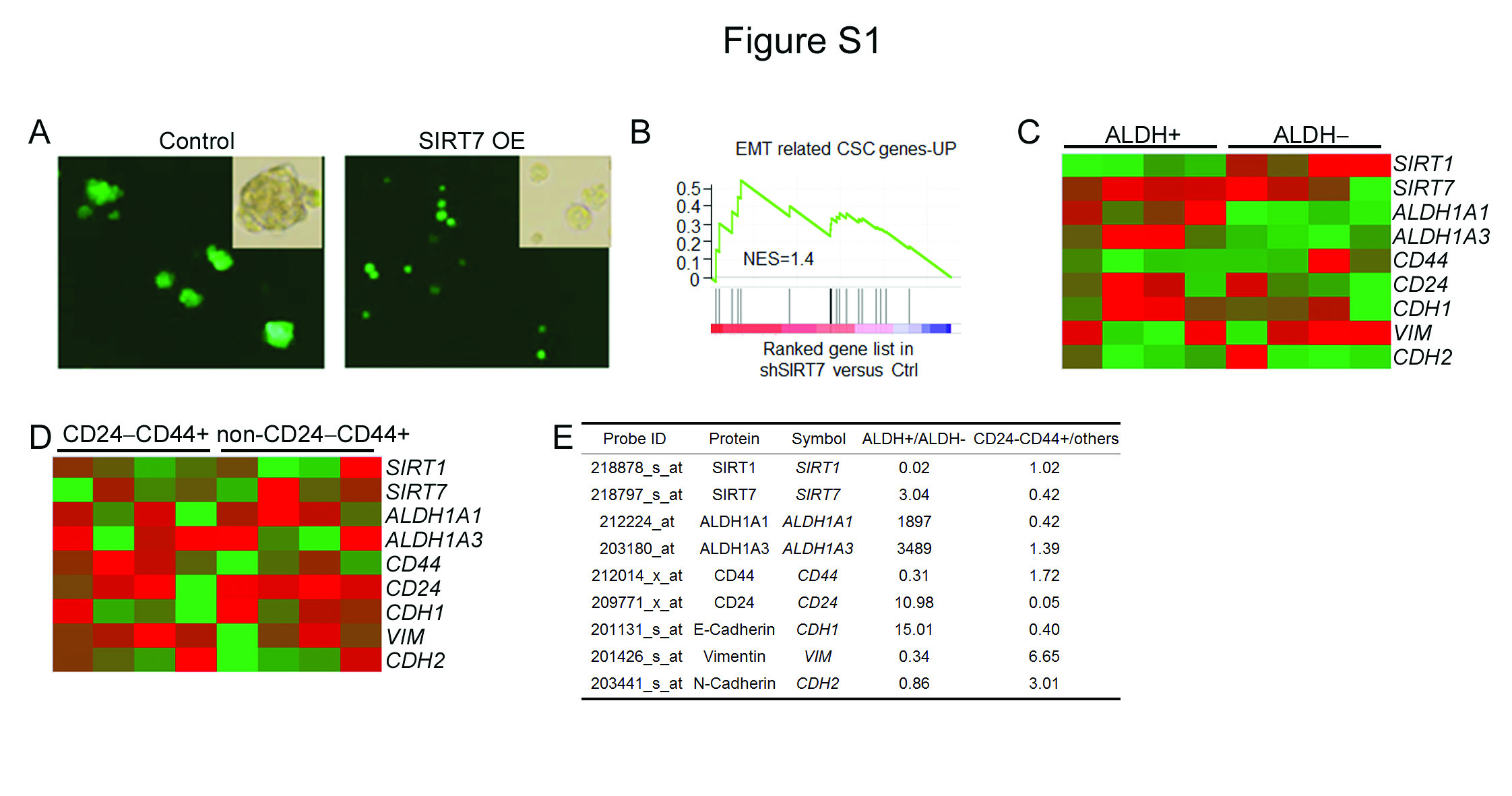

Supplement: Supplementary file 2 — Supplementary Figure S1 [file 41388_2018_370_MOESM2_ESM.jpg]

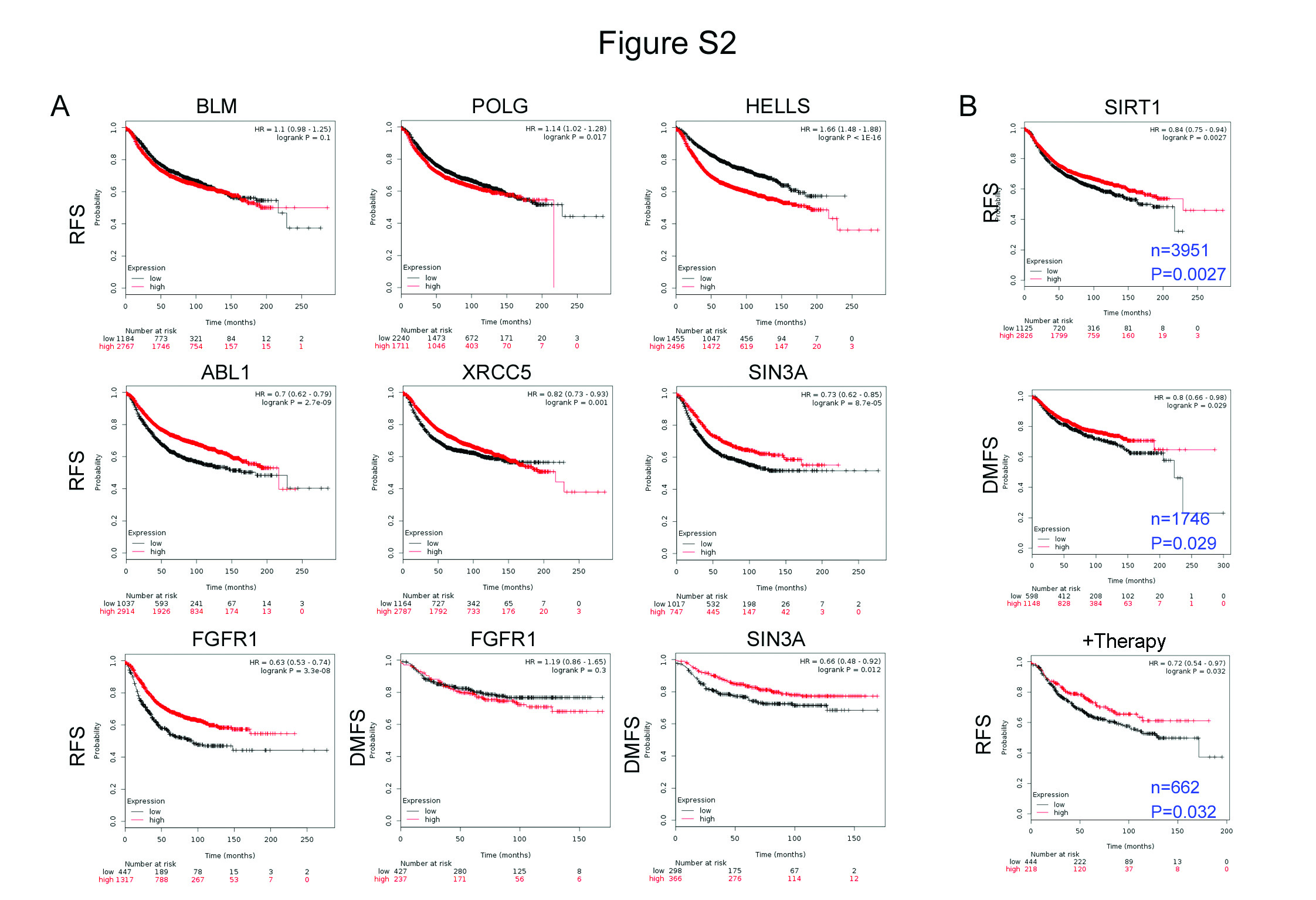

Supplement: Supplementary file 3 — Supplementary Figure S2 [file 41388_2018_370_MOESM3_ESM.jpg]

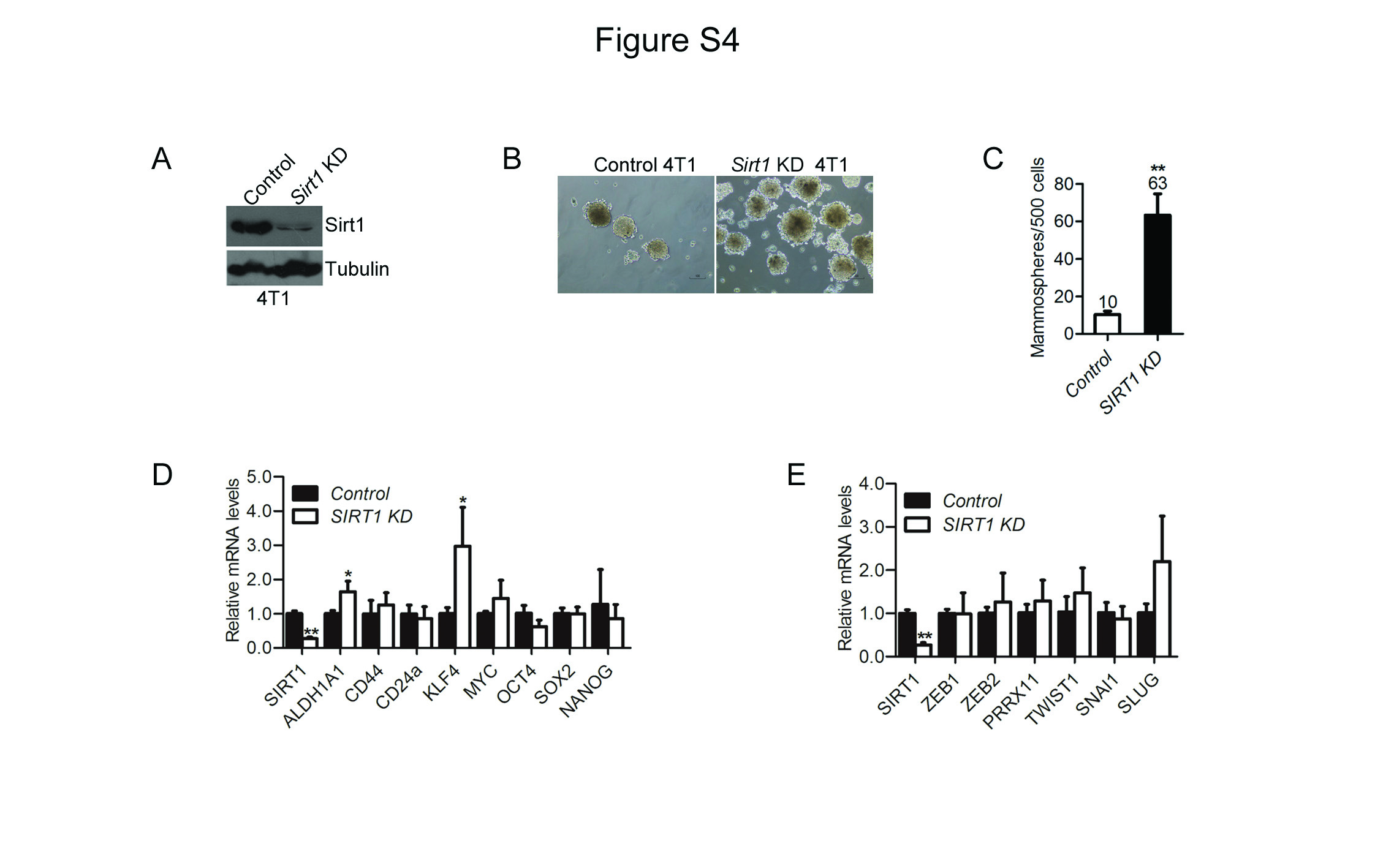

Supplement: Supplementary file 4 — Supplementary Figure S3 [file 41388_2018_370_MOESM4_ESM.jpg]

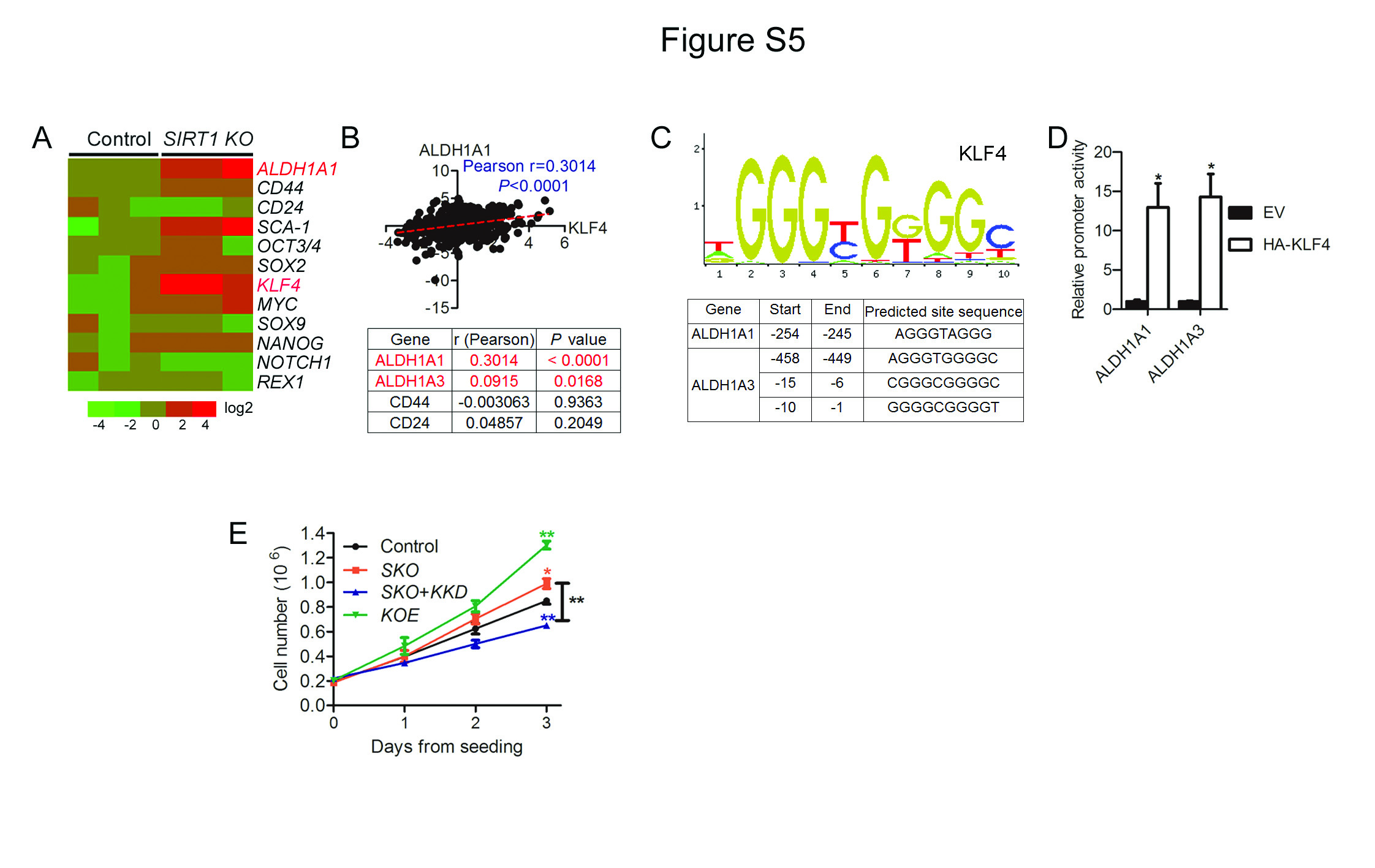

Supplement: Supplementary file 5 — Supplementary Figure S4 [file 41388_2018_370_MOESM5_ESM.jpg]

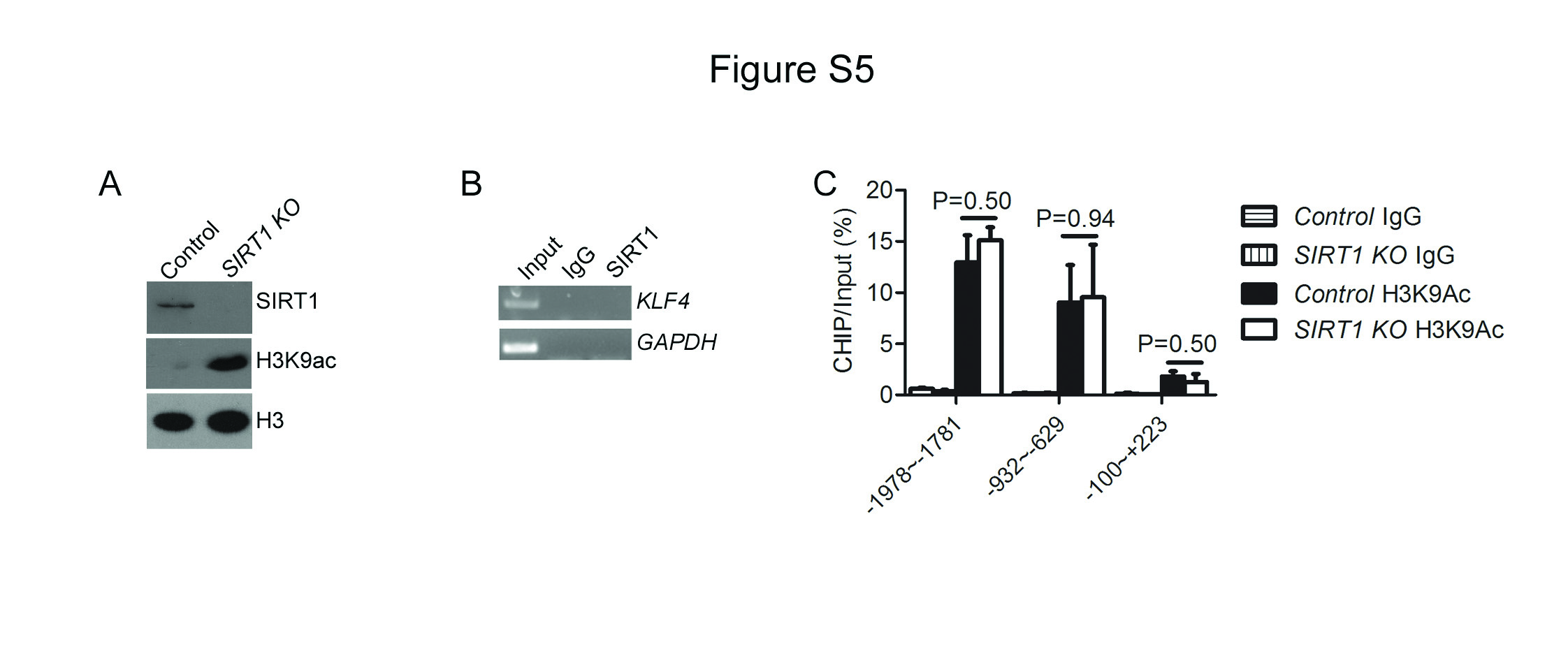

Supplement: Supplementary file 6 — Supplementary Figure S5 [file 41388_2018_370_MOESM6_ESM.jpg]

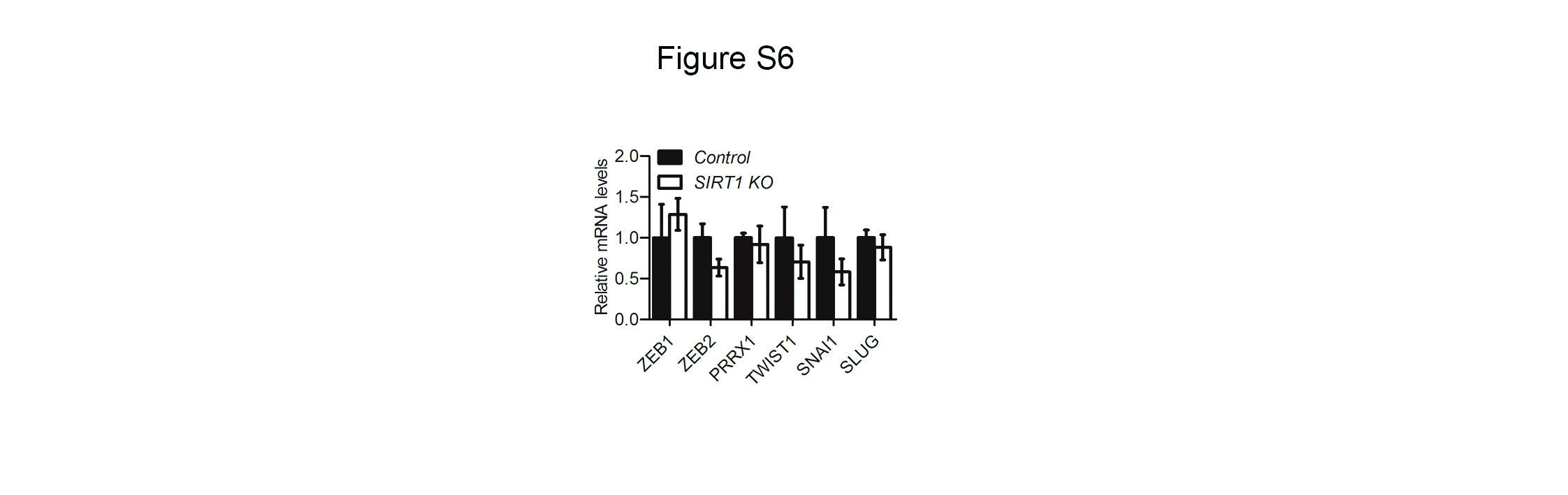

Supplement: Supplementary file 7 — Supplementary Figure S6 [file 41388_2018_370_MOESM7_ESM.jpg]

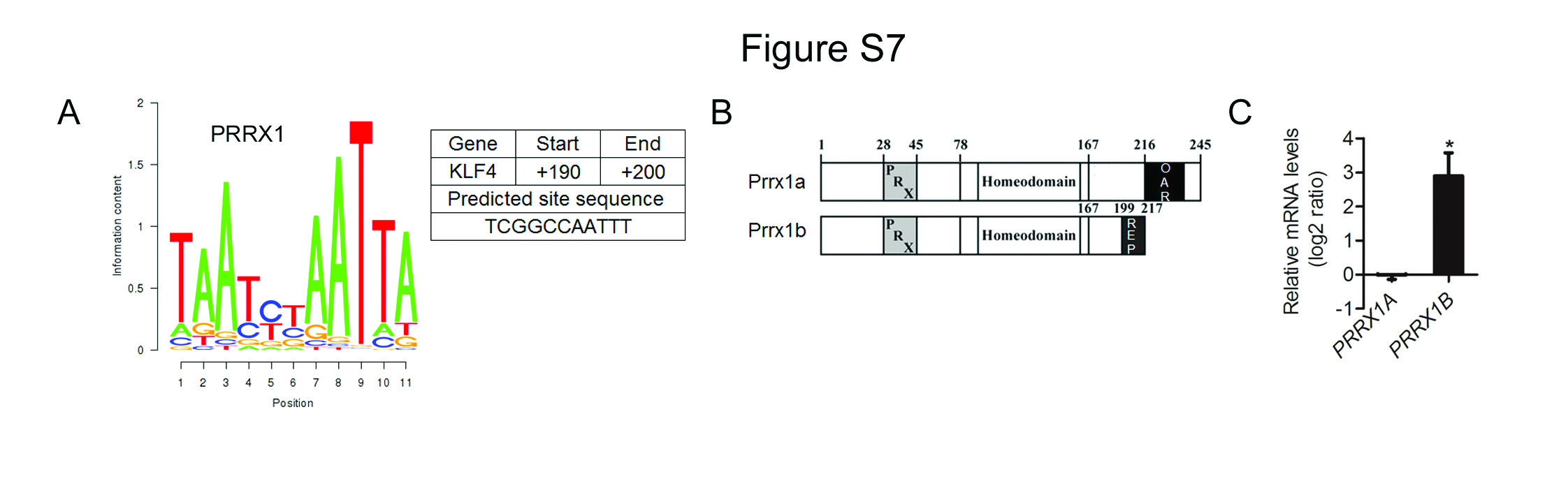

Supplement: Supplementary file 8 — Supplementary Figure S7 [file 41388_2018_370_MOESM8_ESM.jpg]

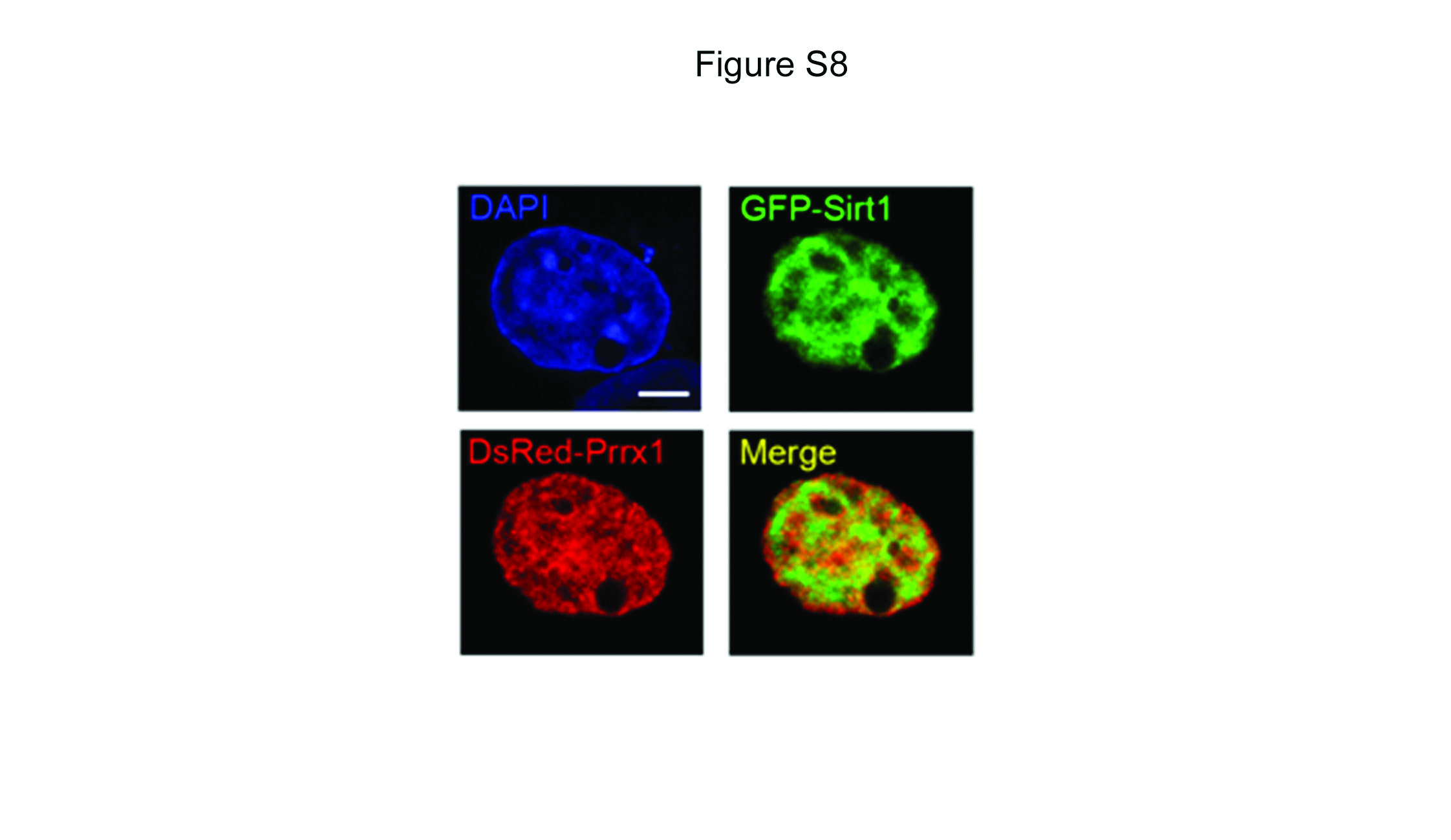

Supplement: Supplementary file 9 — Supplementary Figure S8 [file 41388_2018_370_MOESM9_ESM.jpg]

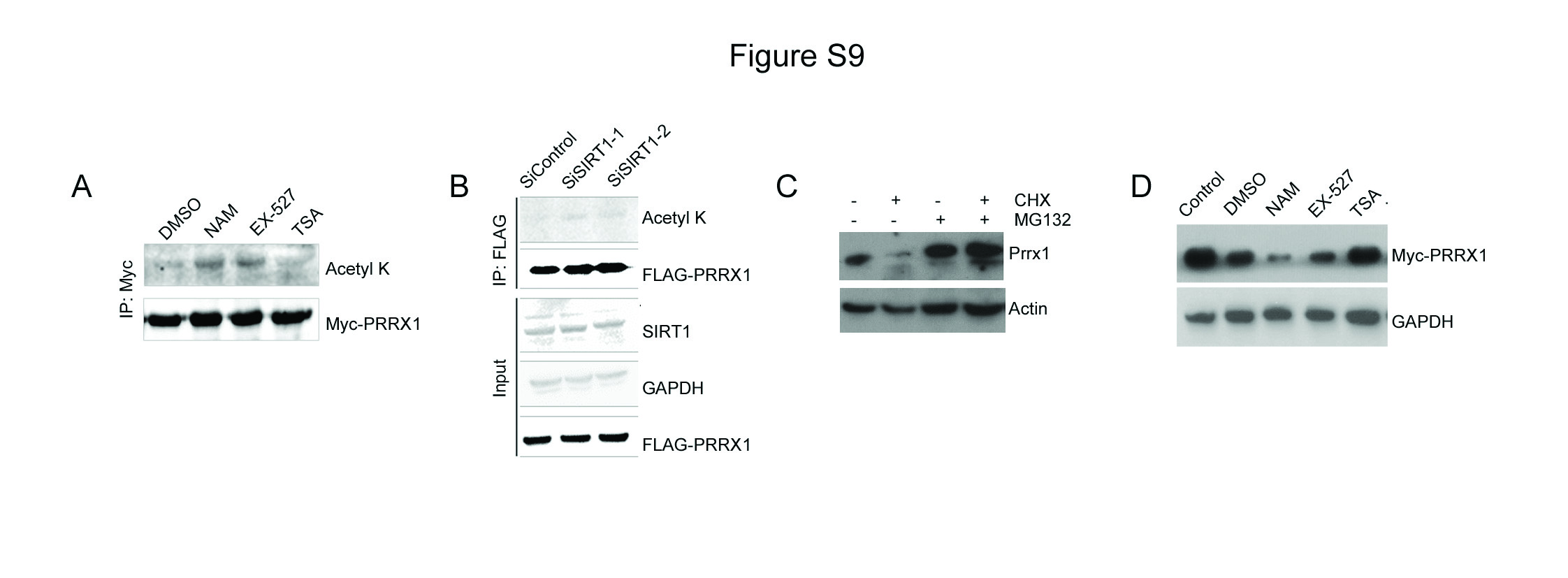

Supplement: Supplementary file 10 — Supplementary Figure S9 [file 41388_2018_370_MOESM10_ESM.jpg]

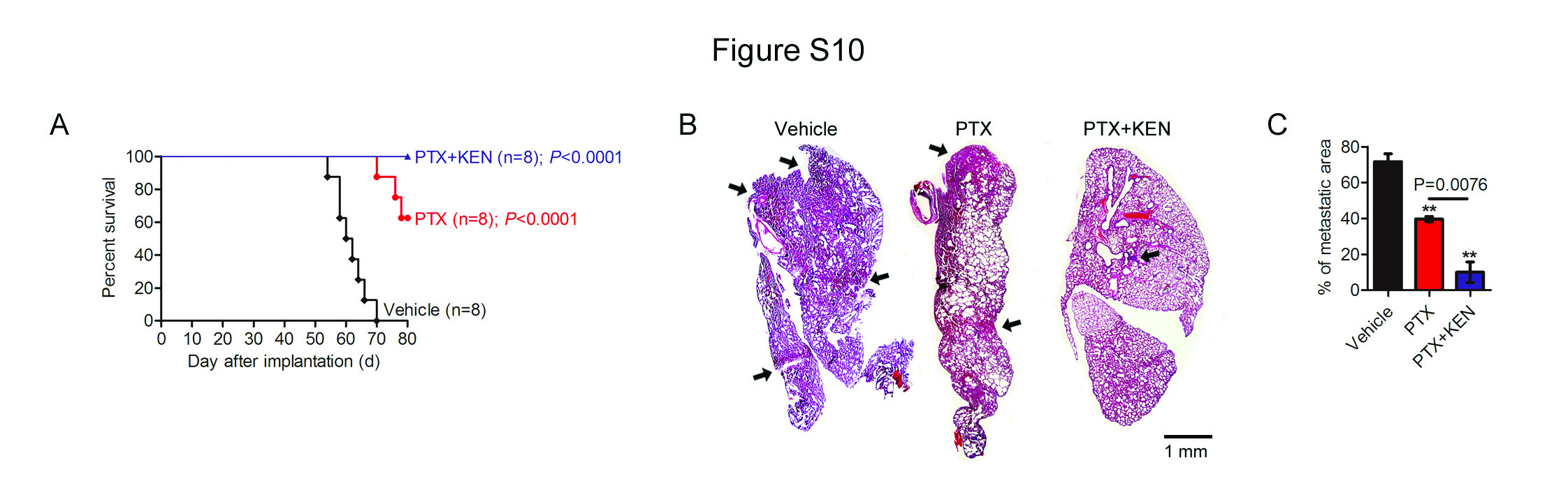

Supplement: Supplementary file 11 — Supplementary Figure S10 [file 41388_2018_370_MOESM11_ESM.jpg]

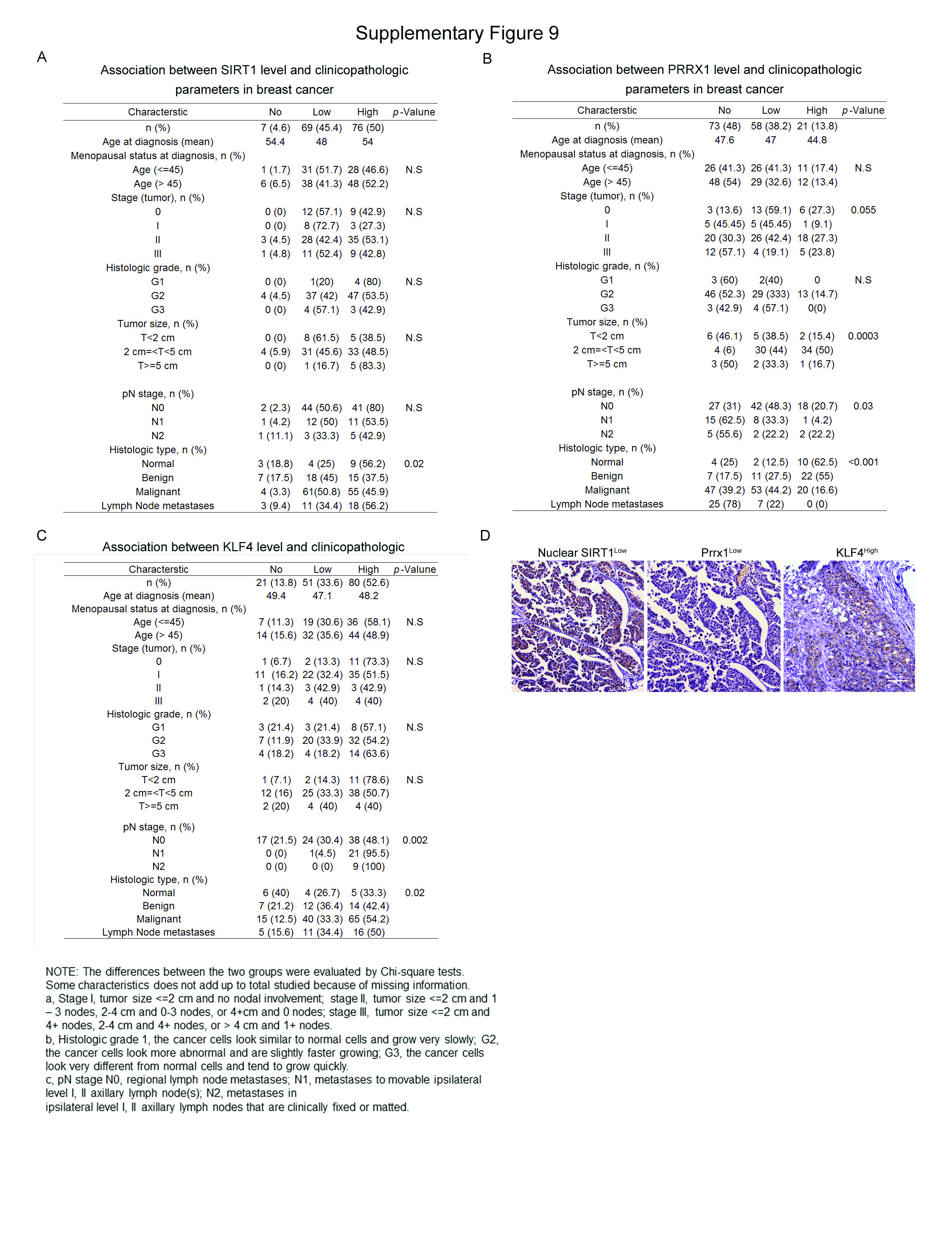

Supplement: Supplementary file 12 — Supplementary Figure S11 [file 41388_2018_370_MOESM12_ESM.jpg]
